# Galectin-3 favours tumour metastasis via the activation of β-catenin signalling in hepatocellular carcinoma

**DOI:** 10.1038/s41416-020-1022-4

**Published:** 2020-08-17

**Authors:** Mengjia Song, Qiuzhong Pan, Jieying Yang, Junyi He, Jianxiong Zeng, Shaoyan Cheng, Yue Huang, Zi-Qi Zhou, Qian Zhu, Chaopin Yang, Yulong Han, Yan Tang, Hao Chen, De-Sheng Weng, Jian-Chuan Xia

**Affiliations:** 1 Department of Biotherapy, Sun Yat-sen University Cancer Center, Guangzhou, P. R. China; 2 Collaborative Innovation Center for Cancer Medicine, State Key Laboratory of Oncology in South China, Sun Yat-sen University Cancer Center, Guangzhou, China; 3 State Key Lab of Molecular Oncology & Immunology Department, National Cancer Center/National Clinical Research Center for Cancer/Cancer Hospital, Chinese Academy of Medical Sciences and Peking Union Medical College, 100021 Beijing, China

**Keywords:** Metastasis, Oncogenes, Tumour angiogenesis, Cell signalling

## Abstract

**Background:**

High probability of metastasis limited the long-term survival of patients with hepatocellular carcinoma (HCC). Our previous study revealed that Galectin-3 was closely associated with poor prognosis in HCC patients.

**Methods:**

The effects of Galectin-3 on tumour metastasis were investigated in vitro and in vivo, and the underlying biological and molecular mechanisms involved in this process were evaluated.

**Results:**

Galectin-3 showed a close correlation with vascular invasion and poor survival in a large-scale study in HCC patients from multiple sets. Galectin-3 was significantly involved in diverse metastasis-related processes in HCC cells, such as angiogenesis and epithelial-to-mesenchymal transition (EMT). Mechanistically, Galectin-3 activated the PI3K-Akt-GSK-3β-β-catenin signalling cascade; the β-catenin/TCF4 transcriptional complex directly targeted IGFBP3 and vimentin to regulate angiogenesis and EMT, respectively. In animal models, Galectin-3 enhanced the tumorigenesis and metastasis of HCC cells via β-catenin signalling. Moreover, molecular deletion of Galectin-3-β-catenin signalling synergistically improved the antitumour effect of sorafenib.

**Conclusions:**

The Galectin-3-β-catenin-IGFBP3/vimentin signalling cascade was determined as a central mechanism controlling HCC metastasis, providing possible biomarkers for predicating vascular metastasis and sorafenib resistance, as well as potential therapeutic targets for the treatment of HCC patients.

## Background

As one of the most common cancers, hepatocellular carcinoma (HCC) is the second leading cause of cancer-related death worldwide, with ~50% of the total cases and deaths occurring in China.^1^ Despite recent advances in therapeutic strategies, the high probability of metastasis limits long-term survival, even after receiving potentially curative treatment.^2^ Anti-angiogenic therapies for the treatment of HCC have been well established in recent years, among which, sorafenib is globally approved as a second-line treatment for unresectable or metastatic HCC.^3,4^ However, sorafenib only improves the survival of HCC patients by an average of 3 months,^5^ and some patients demonstrate sorafenib resistance due to metastasis.^6,7^ Tumour metastasis is a multifactorial and multistage process involving dissemination, invasion, intravasation, survival, extravasation and colonisation, which are modulated by a series of inter and intracellular signalling pathways.^8^ To date, it is an urgency to unveil the unknown molecular mechanisms underlying HCC metastasis.

Epithelial-to-mesenchymal transition (EMT) plays a key role in the early phase of metastasis, during which cells lose cell–cell interactions, due to the lack of E-cadherin expression, and acquire increased motility, invasiveness and extracellular matrix-degrading capability.^9–11^ Aberrations in a diverse set of transcription factors and microRNAs causing EMT-dependent metastasis have been highlighted in HCC.^12–15^ In addition to EMT, tumour angiogenesis is an essential process for metastasis;^3,16^ since the tumour microvasculature is insufficient, chaotic and leaky, it provides an effective transport system for tumour cell dissemination to distant sites.^3,16^ Angiogenic pathways, such as vascular endothelial growth factor (VEGF)/VEGF receptor (VEGFR), platelet-derived growth factor (PDGF)/PDGF receptor (PDGFR), fibroblast growth factor (FGF)/FGF receptor (FGFR), angiopoietin/Tie and Endoglin (CD105) signalling,^3^ have also been demonstrated to be dysregulated in HCC. In fact, both EMT and angiogenesis are complex processes with the potential involvement of multiple signalling pathways; therefore, a better understanding on molecular underpinnings of them will allow more progress in metastatic HCC-target therapy.

Galectin-3, a multifunctional protein encoded by *LGALS3*, belongs to the β-galactoside-binding protein family. As a cytosolic protein, Galectin-3 can easily cross the intracellular and plasma membranes into the nucleus and mitochondria or be externalised, regulating several biological processes.^17^ Numerous studies have indicated that Galectin-3 plays a significant role in tumour progression in distinct manners, such as angiogenesis, chemo-resistance, immunosuppression and metastasis.^18,19^ In addition, activation of β-catenin pathway by Galectin-3 has been reported in multiple cancer types including breast, lung, colon cancers, leukaemia and sarcomas.^17,20^ Our previous study showed that Galectin-3 was a prognostic factor in patients with HCC,^21^ indicating that Galectin-3 may be a crucial regulator of HCC progression, even the metastatic process. In the present study, we aimed to comprehensively investigate whether Galectin-3 is involved in the metastasis of HCC and evaluate the possible molecular mechanisms underlying this process.

## Methods

### Patients and samples

For real-time quantitative reverse transcription-polymerase chain reaction (qPCR), HCC tissues and adjacent normal tissues (*n* = 60) were obtained from patients with primary HCC at the Sun Yat-sen University Cancer Center from 2005 and 2008. After resection, fresh tissues were immediately immersed in RNAlater® (Ambion, Austin, TX, USA), incubated overnight at 4 °C, and subsequently stored at −80 °C until RNA isolation. For immunohistochemistry (IHC) or immunofluorescence (IF) staining, the training set, a total of 287 HCC tissues were collected at the Cancer Center of Sun Yat-sen University from 2005 to 2008. Patients with tumour node metastasis (TNM) stage III received postoperative adjuvant sorafenib therapy. The criterion for sorafenib resistance is that CT scans show the progression of tumour lesions after treatment for more than 1 month. The criterion for sorafenib sensitivity is that CT scans show the tumour lesions regress or are stable after treatment for more than 1 month, and this phenomenon lasted for more than 4 weeks. The testing set was an HCC tissue microarray purchased from Outdo Biotechnology Company (Outdo Biotechnology, Shanghai, China), which included 90 pairs of cancerous and adjacent non-cancerous tissues. These tissues in this set were collected between June 2007 and November 2008. None of the patients in these two sets received no prior anticancer treatments. All the tissue samples in the two sets were collected within 30 min after operation and snap-frozen in liquid nitrogen.

### Animal models

All animal procedures complied with the ARRIVE guidelines and were carried out according to the National Institutes of Health guide for the care and use of Laboratory animals. All cells used in in vivo study were stably expressed luciferase. To explore the effect of Galectin-3 on tumorigenesis and the role of β-catenin in this process in mice, forty 6-week-old female BALB/c nude mice (Vital River Laboratory Animal Technology Co. Ltd, Beijing, China) were randomly divided into eight groups (*n* = 5 each) and hypodermically injected with Hep3B-vector, Hep3B-Galectin-3, Hep3B-Galectin-3-shControl, Hep3B-Galectin-3-shβ-catenin, Huh7-shControl, Huh7-shGalectin3, Huh7-shGalectin-3-vector and Huh7-shGalectin-3-β-catenin (5 × 10^6^ cells in 100 μL PBS). Tumour growth was monitored once per week using an in vivo imaging system (PerkinElmer, IVISLumina Series III, USA) at day 7, 14 and 21. Mice were killed by cervical dislocation after being anaesthetised with 10% chloral hydrate at day 22. All the xenografts were harvested, and tumour weight was measured. IHC staining was performed to examine the expression of CD34, IGFBP3 and vimentin.

For metastasis model construction, forty 6-week-old female BALB/c nude mice were randomly divided into eight groups (*n* = 5 each) and injected with Hep3B-vector, Hep3B-Galectin-3, Hep3B-Galectin-3-shControl, Hep3B-Galectin-3-shβ-catenin, Huh7-shControl, Huh7-shGalectin-3, Huh7-shGalectin-3-vector and Huh7-shGalectin-3-β-catenin (2.5 × 10^6^ cells in 100 μL PBS) via tail vein injection. Tumour metastasis was monitored once per week using an in vivo imaging system at day 14, 21 and 28. All mice were killed by cervical dislocation after being anaesthetised with 10% chloral hydrate at day 29, and lung weight was used to measure the lung metastasis. H&E staining was performed to examine the numbers of micro-metastatic pulmonary nodules.

To assess whether blockade of Galectin-3-β-catenin signalling has an effect on the sensitivity of HCC cells to sorafenib, forty 6-week-old female BALB/c nude mice were randomly divided into four groups (*n* = 10 each) and hypodermically injected with Huh7-shControl, Huh7-shGalectin-3, Huh7-shGalectin-3-vector and Huh7-shGalectin-3-β-catenin (5 × 10^6^ cells in 100 μL PBS). Each group was randomly dived into two groups (a total of eight groups, *n* = 5 each) at day 7. DMSO or sorafenib (Sigma–Aldrich) was orally administrated (in drinking water, 0.3 mg/mL, 3–4 mL/mouse/day) until day 28. Tumour growth was monitored once per week using an in vivo imaging system from day 12 to day 26. All mice were killed by cervical dislocation after being anaesthetised with 10% chloral hydrate at day 27. Xenografts were harvested and tumour weight was measured.

For the quantification of photo flux in animal models, luciferin (150 mg/kg body weight) kinetic curves were firstly performed to determine peak signal time of for the tumour growth and metastasis mouse models by using in vivo imaging system, which were about at 10 min and 15 min after intraperitoneal injection of luciferin, respectively. Then, the luminescence of each mouse in tumour growth and metastasis models were recorded at 10 and 15 min after luciferin injection, respectively. Finally, the total photo flux of each mouse in all sets were quantified by Living Image software.

### IHC and IF staining

For IHC staining, formalin-fixed paraffin-embedded sections were deparaffinised in xylene, hydrated with ethanol gradient, and washed briefly in tap water. Endogenous peroxidase was blocked with methanol containing 0.3% hydrogen peroxide for 30 min. To restore antigenicity, slices were boiled in 10 mM citrate buffer (pH 5.8) in a microwave oven for 30 min. Next, the sections were incubated with goat serum diluted in PBS at 22 °C for 30 min. Subsequently, the sections were incubated with BATF3 specific primary antibody diluted 1:200 at 4 °C overnight (Abcam, Cambridge, UK). The next day, the sections were washed with fresh PBS and incubated with horseradish peroxidase-conjugated secondary antibody at room temperature for 30 min. Finally, the sections were stained with 3,30 diaminobenzidine (DAB) substrate (Dako, Carpinteria, CA, USA) and counterstained with Mayer haematoxylin. Five random fields per sample were assessed using a light microscope (Olympus, Tokyo, Japan). The intensity of the IHC staining was evaluated using the following criteria: 0, negative; 1, low; 2, medium and 3, high. The extent of IHC staining was scored as 0, 0–10% stained; 1, 10–40% stained; 2, 40–70% stained and 3, 70–100% stained. The final scores were calculated by multiplying the scores of the intensity by those of the extent. All samples in each set were divided into four grades according to IHC score: 0, −; 1–3, +; 4–6, ++ and 7–9, +++. Furthermore, “−” and “+” were grouped into “low expression”, while “++ and + ++” were grouped into “high expression”.

For cell IF staining, cells were fixed in 4% paraformaldehyde for 15 min, washed twice with cold PBS and then incubated with antibody at 4 °C overnight. After washing with PBS, the cells were incubated with an appropriate fluorescent dye-conjugated secondary antibody for 1 h, and then nuclear staining with DAPI was performed for 10 min. Observe the cells under a fluorescent microscope.

Tissue IF staining was performed using a Pano-Panel Kit according to the manufacturer’s instructions (Panovue, Beijing, China). Tissues or cells were examined for the expression of Galectin-3, β-catenin, CD34, IGFBP3 or vimentin. Images of IF staining were taken and quantitatively analysed using tissue microarray scanner Vectra 2 software (Perkin Elmer, Waltham, Massachusetts, USA).

The following rabbit anti-human primary antibodies were used for IHC and IF staining: Galectin-3 (Cell Signaling Technology, Boston, MA, USA), β-catenin (Cell Signaling Technology), CD34 (Abcam, Cambridge, MA, USA), IGFBP3 (Abcam) and vimentin (Proteintech Group, Chicago, Illinois, USA).

### Western blotting

The protocol used for western blotting is described elsewhere.^22^ Total cellular protein was extracted in lysis buffer by radioimmunoprecipitation assay. The protein concentration was determined by using the bicinchoninic acid kit (Biyuntian, Jiangsu, China). Load the same amount of protein onto 10% SDS-polyacrylamide gel electrophoresis gel. After electrophoresis, the protein was blotted onto a polyvinylidene fluoride membrane. The membrane was blocked in 5% skim milk and incubated with the primary antibody overnight at 4 °C, and then treated with secondary antibody at 37 °C for 2 h. The primary rabbit anti-human antibodies used were as follows: ECAD (Cell Signaling Technology), NCAD (Cell Signaling Technology), vimentin (Proteintech Group), MMP1 (Cell Signaling Technology), MMP3 (Abcam), IGFBP3 (Abcam), β-catenin (Cell Signaling Technology), Akt (Cell Signaling Technology), phospho-Akt (Cell Signaling Technology), GSK-3β (Cell Signaling Technology), phospho-GSK-3β (Cell Signaling Technology), TBP (Proteintech Group) and β-actin (Cell Signaling Technology).

### Chromatin immunoprecipitation (ChIP) assay

The ChIP assay was performed according to the manufacturer’s instructions (Cell Signaling Technology). Anti-β-catenin (Cell Signaling Technology) and anti-TCF4 (Cell Signaling Technology) antibodies were used to immunoprecipitate the chromatin in HCC cells. qPCR was performed using primers identified for the TCF4-binding site in the *IGFBP3* and *VIM* promoter region as follows: 5′-TGTTACTTGTCAAAGCCACTTT-3′ and 5′-ATAGTCTCCTTTTATCTCCCTTG-3′.

### The Cancer Genome Atlas (TCGA) database analysis

The TCGA dataset for HCC was obtained from the UCSC Cancer Browser (https://genome-cancer.ucsc.edu). Gene expression levels based on the mRNA sequencing data of TCGA are shown as the mean ± standard error of the mean (SEM) of triplicate determinations. Survival, Gene Ontology (GO), Gene Set Enrichment (GSEA) and correlation analyses in HCC patients were performed according to the TCGA mRNA sequencing data and follow-up information. All analyses were performed using the R Package ‘Survival’.

### Statistical analysis

Seventeen Data are shown as the mean ± S.D. and were analysed using the Student’s *t*-test or χ^2^ test. A paired *t*-test was used for paired samples. The Kaplan–Meier method was used to plot survival curves. The log-rank test was used to analyse the difference in survival time among the patient subgroups. Cox’s proportional hazards regression model was used to assess the prognostic value of the risk factors. Statistical analyses were performed using GraphPad Prism 7 and SPSS software (GraphPad software, La Jolla, CA, USA). *P* < 0.05 was considered statistically significant. Other materials and methods used in the present study are listed in Supplementary Materials and Methods.

## Results

### A high expression level of Galectin-3 is closely correlated with vascular invasion and poor survival in HCC patients

**Fig. 1:**
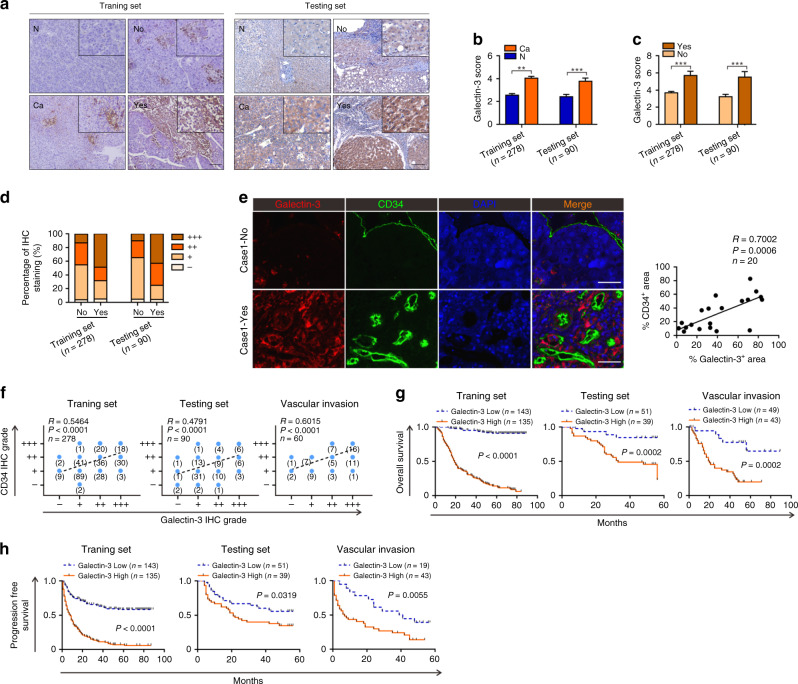
A high level of Galectin-3 expression is significantly associated with vascular invasion and poor survival in HCC patients. **a**−**c** IHC staining shows the expression level of Galectin-3 in HCC tissues and paired adjacent normal tissues, as well as in HCC tissues with or without vascular invasion in the training and testing sets. Scale bars, 100 μm. **d** The percentage of Galectin-3 IHC staining grade in patients with or without vascular invasion in the training and testing sets. **e** Tissue IF staining shows the co-expression pattern of Galectin-3 (red) and CD34 (green) in the HCC tumour microenvironment. DAPI, blue. Figure panel pairs representative images taken with different zooming options; Scale bars, 100 μm. **f** Spearman’s correlation analysis of the association between Galectin-3 and CD34 in the training (left), testing (middle), and vascular invasion (right) sets. **g, h** Survival analyses between “Galectin-3 high” and “Galectin-3 low” groups for the overall survival (**g**) and progression-free survival (**h**) based on IHC staining in the training (left), testing (middle), and vascular invasion (right) sets. N normal, Ca cancer, No without vascular invasion, Yes with vascular invasion. The results represent three independent experiments. **P < 0.01; ***P < 0.001.

Our previous study showed that Galectin-3 plays an important role in HCC progression.^21^ In the present study, we constructed a large-scale model of HCC by increasing the number of cases to 278 (training set, *n* = 278) and obtaining an HCC tissue microarray consisting of a further 90 cases (testing set, *n* = 90) for validation. In both selected sets, mitotic index has been demonstrated as a vital prognostic biomarker of tumour progression and metastasis (Supplementary Fig. 1) according to the previously reported method.^23^ To determine the expression level of Galectin-3 and its clinical significance in HCC, we analysed HCC tissues and paired adjacent normal tissues using qPCR and IHC staining. The results demonstrate that Galectin-3 was highly expressed in HCC tissues at both the mRNA (Supplementary Fig. 2a, b) and protein (Fig. 1a, b). Interestingly, we found that cases with vascular invasion had a higher expression level than those without vascular invasion (Supplementary Fig. 2c; Fig. 1a, c). Accordingly, a larger percentage of cases with a high Galectin-3 IHC staining score occurred in patients with vascular invasion in both the training and testing sets (Fig. 1d).

Subsequently, we examined the co-expression pattern of Galectin-3 and CD34, a microvascular marker, in HCC tissues by IF staining. The results indicated a positive correlation between Galectin-3 and microvascular in HCC tissues (Fig. 1e), suggesting that increased expression of Galectin-3 expression may be involved in the process of tumour angiogenesis in the HCC microenvironment. To verify this hypothesis, cases with vascular invasion in the two sets were screened out to construct a “vascular invasion” set (*n* = 62). We further analysed the correlation between Galectin-3 and CD34 expression based on their IHC staining scores in the HCC samples in the training, testing and vascular invasion sets. As expected, a strong correlation between the expression of these two proteins was exhibited in all three sets, especially in the vascular invasion set (Fig. 1f).

For the clinical relevance of Galectin-3, we also found a close relationship between a high Galectin-3 expression level and tumour size, tumour number, TNM stage and vascular invasion in the training and testing sets, in addition to a strong association with tumour number and TNM stage in the vascular invasion set (Table 1). Survival analysis revealed that a high expression level of Galectin-3 was significantly correlated with poor overall survival (OS) and progression-free survival (PFS) in the training, testing and vascular invasion sets (Fig. 1g, h). Similar results were observed by qPCR (Supplementary Fig. 2d, e) and in the TCGA dataset (Supplementary Fig. 2f) based on survival analysis. We also constructed the set of nonvascular invasion and found that high Galectin-3 was also associated with lower OS and PFS rates in this set (Supplementary Fig. 3), indicating that Galectin-3 was also a high-risk prognostic factor in patients with nonvascular invasion. Moreover, univariate and multivariate Cox regression analyses identified Galectin-3 as an independent prognostic factor for OS and PFS in all three sets (Table 2). To assess its discriminatory ability, we performed receiver operating characteristic (ROC) analysis of all three sets. According to the area under the curve (AUC), Galectin-3 had a good sensitivity and specificity for predicting survival in HCC patients, particularly OS, for which all AUC values were above 0.75 (Supplementary Fig. 4a, b). Additionally, Galectin-3 showed good performance in predicting vascular invasion in the training and testing sets, with AUC values above 0.75 (Supplementary Fig. 4c). Taken together, these results suggest that Galectin-3 is a clinically noteworthy protein that may favour HCC progression via the regulation of vascular invasion-driven metastasis.

**Table 1:** Association of Galectin-3 expression with clinicopathological features in patients with HCC from training, testing and vascular invasion sets.

	Training set (*n* = 278)					Testing set (*n* = 90)					Vascular invasion (*n* = 62)				
		Galectin-3 expression (*n* = 278)					Galectin-3 expression (*n* = 90)					Galectin-3 expression (*n* = 62)			
Characteristic	Total	Low *n* = 143 (51.4%)	High *n* = 135 (48.6%)	*χ*^*2*^	*P*-value	Total	Low *n* = 51 (56.7%)	High *n* = 39 (43.3%)	*χ*^*2*^	*P*-value	Total	Low *n* = 19 (30.6%)	High *n* = 43 (69.4%)	*χ*^*2*^	*P*-value
Age, years															
≥50	121	65	56	0.4459	0.5043	53	27	26	1.72	0.1897	38	14	24	1.77	0.1829
<50	157	78	79			37	24	13			24	5	19		
Gender															
Male	232	121	111	0.288	0.5915	10	6	4	0.05	0.8215	34	7	27	3.58	0.0584
Female	46	22	24			80	45	35			28	12	16		
HBsAg															
Positive	245	124	121	0.5646	0.4524	70	40	30	0.03	0.8646	50	13	37	2.62	0.1053
Negative	33	19	14			20	11	9			12	6	6		
Liver cirrhosis															
Yes	175	82	93	3.969	**0.0463**	80	47	33	1.27	0.2593	48	18	30	3.38	0.0660
No	103	61	42			10	4	6			14	1	13		
Tumour size															
≥5 cm	165	70	95	13.21	**0.0003**	35	14	21	6.48	**0.0109**	43	10	33	3.60	0.0576
<5 cm	113	73	40			55	37	18			19	9	10		
Tumour number															
Multiple	105	43	62	7.428	**0.0064**	11	6	5	0.02	0.8796	20	2	20	7.45	**0.0063**
Single	173	100	73			79	45	34			40	17	23		
AFP															
≥400 ng/ml	119	63	56	0.188	0.6646	32	20	12	0.69	0.4068	29	7	22	1.09	0.2975
<400 ng/ml	159	80	79			58	31	27			33	12	21		
Histological differentiation															
Well	50	30	20	1.789	0.1811	34	24	10	4.31	**0.0378**	33	11	22	0.24	0.6243
Poor	228	113	115			56	27	29			29	8	21		
Tumour encapsulation															
Yes	157	77	80	0.8278	0.3629	43	26	27	0.48	0.4867	36	11	25	0.0003	0.9856
No	121	66	55			47	25	22			26	8	18		
TNM stage															
I	174	105	69	15.45	**<0.0001**	58	39	19	7.43	**0.0064**	41	13	28	7.06	**0.7999**
II or III	103	37	66			32	12	20			21	6	15		
Vascular invasion															
Yes	41	13	28	7.496	**0.0062**	21	6	15	8.80	**0.0030**					
No	237	130	107			69	45	24							

Statistically significant values are in bold.

**Table 2:** Univariate and multivariate analysis of OS and PFS in patients with HCC from traning, testing and vascular invasion sets.

Variable	Traning set (*n* = 278)								Testing set (*n* = 90)								Vascular invasion (*n* = 62)							
	Overall survival				Progression-free survival				Overall survival				Progression-free survival				Overall survival				Progression-free survival			
	Univariate cox		Multivariate cox		Univariate cox		Multivariate cox		Univariate cox		Multivariate cox		Univariate cox		Multivariate cox		Univariate cox		Multivariate cox		Univariate cox		Multivariate cox	
	*P*-value	HR (95% CI)	*P*-value	HR (95% CI)	*P*-value	HR (95% CI)	*P*-value	HR (95% CI)	*P*-value	HR (95% CI)	*P*-value	HR (95% CI)	*P*-value	HR (95% CI)	*P*-value	HR (95% CI)	*P*-value	HR (95% CI)	*P*-value	HR (95% CI)	*P*-value	HR (95% CI)	*P*-value	HR (95% CI)
*Age* ≥ 50 or <50	0.441	0.87 (0.62–1.23)			0.910	0.98 (0.73–1.32)			0.676	1.17 (0.57–2.38)			0.385	1.29 (0.72–2.31)			0.483	1.28 (0.64–2.53)			0.510	1.2256 (0.67–2.25)		
*Gender* Male or female	0.610	1.13 (0.71–1.80)	**0.027**	1.72 (1.06–2.77)	0.522	0.89 (0.61–1.29)			0.371	0.52 (0.12–2.18)			0.736	0.85 (0.32–2.15)			0.160	1.61 (0.83–3.11)			0.169	1.51 (0.84–2.73)	**0.041**	2.20 (1.03–4.71)
*HBsAg* Positive or negeative	0.672	0.90 (0.54–1.49)			0.383	0.82 (0.53–1.27)			0.720	0.86 (0.38–1.94)			0.914	1.04 (0.53–2.03)			0.406	1.45 (0.60–3.51)			0.350	1.44 (0.67–3.11)		
*Liver cirrhosis* Yes or no	**0.015**	1.59 (1.09–2.31)			0.1716	1.24 (0.91–1.69)			0.732	1.23 (0.37–4.04)			0.440	1.50 (0.54–4.16)			0.675	0.84 (0.36–1.92)			0.951	1.02 (0.47–2.21)		
*Tumour size* ≥ 5 cm or <5 cm	**0.001**	1.84 (1.28–2.64)			**0.001**	1.69 (1.24–2.29)			0.097	1.81 (0.90–3.63)			0.091	1.62 (0.93–2.85)			**0.014**	2.84 (1.24–6.51)			**0.003**	2.97 (1.45–6.12)	**0.009**	2.68 (1.29–5.59)
*TNM stage* II/III or I	**<0.001**	2.46 (1.75–3.46)	**0.004**	1.66 (1.17–2.35)	**<0.001**	2.24 (1.67–3.0)	**<0.001**	1.70 (1.25–2.29)	0.081	1.86 (0.93–3.72)			0.094	1.62 (0.92–2.85)			**0.039**	0.46 (0.22–0.96)			**0.015**	0.44 (0.23–0.85)		
*Tumour encapsulation* Yes or no	0.258	0.82 (0.58–1.16)			0.709	0.95 (0.71–1.27)			0.092	1.85 (0.90–3.79)			0.264	1.38 (0.78–2.43)			0.375	1.35 (0.70–2.62)			0.678	1.13 (0.63–2.05)		
*Tumour number* Multiple or single	**0.003**	1.68 (1.20–2.36)			**0.004**	1.54 (1.15–2.07)	**0.015**	1.45 (1.07–1.96)	0.192	1.81 (0.74–4.42)			0.067	1.97 (0.95–4.08)	**0.025**	2.34 (1.11–4.93)	**<0.001**	4.29 (2.18–8.44)	**0.001**	3.91 (1.93–7.95)	**<0.001**	3.47 (1.87–6.43)	**<0.001**	3.15 (1.68–5.9)
*AFP (ng/ml)* ≥ 400 or <400	0.566	0.90 (0.64–1.28)			0.367	1.14 (0.85–1.53)			0.698	1.15 (0.56–2.36)			0.814	0.93 (0.52–1.68)			0.447	1.29 (0.67–2.48)			0.080	1.70 (0.94–3.07)		
*Histological differeniation* Poor or well	**0.038**	1.67 (1.03–2.72)	**0.017**	1.82 (1.11–2.97)	**0.004**	1.90 (1.23–2.92)	**0.011**	1.75 (1.14–2.70)	**0.003**	4.28 (1.64–11.17)	**0.015**	3.35 (1.27–8.9)	**0.018**	2.15 (1.14–4.07)	**0.018**	2.15 (1.14–4.07)	0.287	1.44 (0.74–2.79)			0.4959	1.23 (0.68–2.21)		
*Vascular invasion* Yes or no	**<0.001**	2.16 (1.41–3.33)			**0.002**	1.83 (1.25–2.68)			0.318	1.46 (0.69–3.09)			0.588	1.19 (0.63–2.25)										
*Galectin-3* High or low	**<0.001**	8.60 (5.90–12.55)	**0.001**	22.77 (12.4–41.8)	**<0.001**	4.86 (3.51–6.74)	**0.003**	4.34 (3.11–6.07)	**0.001**	3.74 (1.77–7.9)	**0.005**	3.00 (1.4–6.44)	**0.037**	1.82 (1.04–3.2)	0.105		**0.001**	5.12 (1.96–13.35)	**0.002**	4.73 (1.75–12.76)	**<0.001**	5.08 (2.72–9.49)	0.085	–

Statistically significant values are in bold.

### Galectin-3 promotes the angiogenesis and EMT of HCC cells

**Fig. 2:**
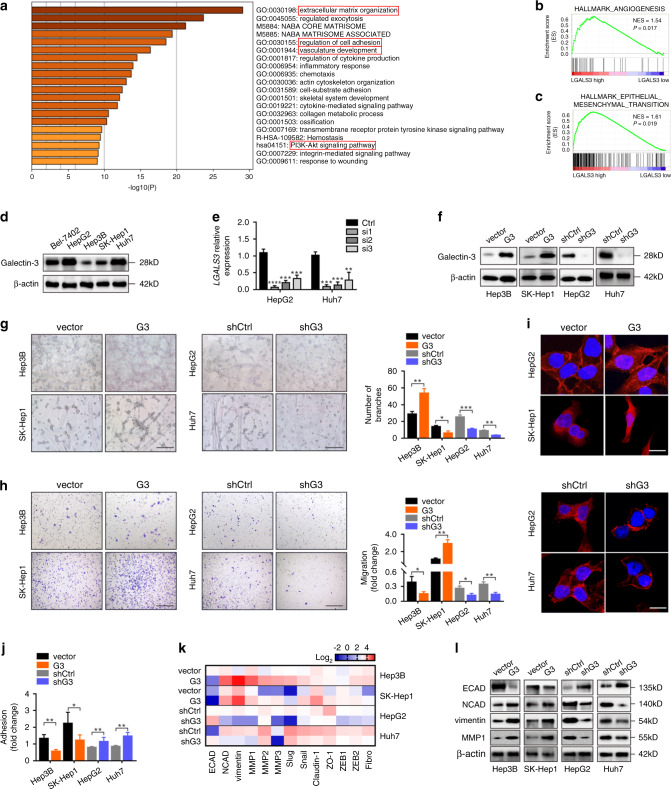
Galectin-3 promotes the angiogenesis and EMT of HCC cells in vitro. **a** GO analysis was performed according to the HCC mRNA sequencing data from TCGA to identify Galectin-3-associated GO terms; the top 20 terms are shown. **b, c** GSEA based on the HCC mRNA sequencing data from TCGA as performed to analyse the correlation between Galectin-3 and angiogenesis (**b**) and EMT (**c**). **d** Western blotting shows the expression of Galectin-3 in HCC cell lines, including Bel-7402, HepG2, Hep3B, SK-Hep1 and Hun7. **e** qPCR shows the mRNA expression of *LGALS3* in Hep3B and SK-Hep1 cells transfected with siControl, siLGALS3#1, siLGALS3#2 or siLGALS3#3. **f** Western blotting shows the expression of Galectin-3 in Hep3B-vector, Hep3B-Galectin-3, SK-Hep1-vector, SK-Hep1-Galectin-3, HepG2-shControl, HepG2-shGalectin-3, Huh7-shControl and Huh7-shGalectin-3 cells. **g**–**j** Tube formation assay (**g**), Transwell migration assay (**h**), IF assay for phalloidin (**i**), and cell adhesion assay (**j**) in Hep3B-vector, Hep3B-Galectin-3, SK-Hep1-vector, SK-Hep1-Galectin-3, HepG2-shControl, HepG2-shGalectin-3, Huh7-shControl and Huh7-shGalectin-3 cells. Scale bars in **g**, **h**, and **i** are 200 μm, 200 μm, and 20 μm, respectively. **k** PCR array shows the expression of EMT-related genes in Hep3B-vector, Hep3B-Galectin-3, SK-Hep1-vector, SK-Hep1-Galectin-3, HepG2-shControl, HepG2-shGalectin-3, Huh7-shControl and Huh7-shGalectin-3 cells. **l** Western blotting shows the expression of ECAD, NCAD, vimentin and MMP1 in Hep3B-vector, Hep3B-Galectin-3, SK-Hep1-vector, SK-Hep1-Galectin-3, HepG2-shControl, HepG2-shGalectin-3, Huh7-shControl and Huh7-shGalectin-3 cells. Ctrl control, G3 Galectin-3. The results represent three independent experiments. *P < 0.05; **P < 0.01; ***P < 0.001.

To identify the biological processes regulated by Galectin-3 during HCC progression, we analysed the HCC mRNA sequencing data from TCGA using GO functional analysis. The results showed that Galectin-3 was significantly related to GO terms, such as extracellular matrix (ECM) organisation, regulation of cell adhesion and vasculature development (Fig. 2a), all of which are metastasis-related processes. Moreover, GSEA, a canonical analysis for the interpretation of genome-wide expression profiles, further proposed Galectin-3 involvement in angiogenesis (Fig. 2b) and EMT (Fig. 2c) gene sets according to TCGA data for HCC.

Next, we examined the expression level of Galectin-3 in five HCC cell lines. According to the results shown in Fig. 2d, we chose Hep3B and SK-Hep1 for the stable overexpression of Galectin-3 since the endogenous expression level was low, while HepG2 and Huh7 were used for stable knockdown since endogenous Galectin-3 expression was high. HepG2 and Huh7 cells were separately infected with three small interfering (si) RNAs against Galectin-3 (Fig. 2e), and the sequence with the highest silencing efficiency was selected for the establishment of stable knockdown cell lines. Galectin-3 expression levels were confirmed in stably overexpressing and knockdown cells (Fig. 2f, Supplementary Fig. 5). Notably, Galectin-3-overexpressing cells harboured greater properties of angiogenesis and migration, while knockdown of Galectin-3 dramatically abrogated these abilities (Fig. 2g, h). Since EMT facilitates the loss of cell–cell interactions and the acquisition of increased motility, invasiveness and ECM-degrading capacity,^9^ we performed an IF assay for phalloidin, an F-actin stain, to assess the effect of Galectin-3 on cell–cell adhesion and mobility. As shown in Fig. 2i, Galectin-3-overexpressed cells displayed many new spike-like protrusions and a more mesenchymal-type morphology at the edges, whereas Galectin-3-knockdown cells showed altered from a spindle-like shape to tight cell-to-cell adhesion as compared with the control cells, suggesting that a high expression level of Galectin-3 is accompanied by the occurrence of EMT. Adhesion assay revealed that Galectin-3 overexpression suppressed cell adhesion ability, while Galectin-3-knockdown could enhance cell adhesion ability (Fig. 2j). Moreover, PCR array analysis identified a cluster of EMT-related genes obviously upregulation by Galectin-3-overexpressed cells but downregulation by Galectin-3-knocked down cells, such as NCAD, vimentin, MMP1, MMP2 and MMP3, as well as a reverse expression of ECAD (Fig. 2k). Consistent with the PCR array results, a similar expression pattern was obtained at the protein level by western blotting (Fig. 2l). Collectively, these results indicate that Galectin-3 promotes the angiogenesis and EMT of HCC cells, which may be potential mechanisms underlying the Galectin-3-mediated metastasis of HCC.

### Activation of the PI3K-Akt-GSK-3β-β-catenin signalling cascade is responsible for the effect of Galectin-3 on the angiogenesis and EMT of HCC cells

**Fig. 3:**
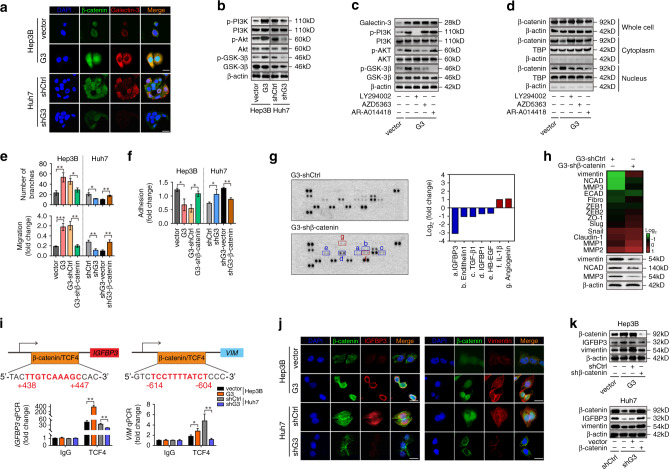
Activation of the PI3K-Akt-GSK-3β-β-catenin signalling cascade is essential for Galectin-3-induced angiogenesis and EMT of HCC cells. **a** IF staining shows the colocalisation of β-catenin (green) and Galectin-3 (red) in Hep3B-vector, Hep3B-Galectin-3, Huh7-shControl and Huh7-shGalectin-3 cells. Scale bars, 20 μm. **b** Western blotting shows the expression of phospho-PI3K, PI3K, phospho-Akt, Akt, phospho-GSK-3β and GSK-3β in Hep3B-vector, Hep3B-Galectin-3, Huh7-shControl and Huh7-shGalectin-3 cells. **c**, **d** Hep3B-Galectin-3 cells were treated with DMSO, PF294002 (20 μmol/L), AZD5363 (10 μmol/L) or AR-A014418 (20 μmol/L). Western blotting shows the expression of Galectin-3, phospho-PI3K, PI3K, phospho-Akt, Akt, phospho-GSK-3β and GSK-3β (**c**). Western blotting of the expression of β-catenin in the whole cell, cytoplasm and nucleus (**d**). **e**, **f** Statistical diagrams of the tube formation assay, Transwell migration assay and cell adhesion assay. **g**, **h** Proteome angiogenesis array shows angiogenesis-related soluble proteins (**g**), and PCR array and western blotting show EMT-related genes/proteins (**h**) in Hep3B-shControl and Hep3B-shβ-catenin cells based on Galectin-3 overexpression. **i** Analysis of the *IGFBP3* and *VIM* promoter identified a TCF4-binding site. Moreover, CHIP was performed using IgG and TCF4 antibodies, followed by qPCR in Hep3B-vector, Hep3B-Galectin-3, Huh7-shControl and Huh7-shGalectin-3 cells. **j** IF staining shows the colocalisation of β-catenin (green) and IGFBP3 (red) (left) or vimentin (red) (right) in Hep3B-vector, Hep3B-Galectin-3, Huh7-shControl and Huh7-shGalectin-3 cells. Scale bars, 20 μm. **k** Western blotting shows the expression of β-catenin, IGFBP3 and vimentin in Hep3B-vector, Hep3B-Galectin-3, Hep3B-Galectin-3-shControl and Hep3B-Galectin-3-shβ-catenin cells (upper) and Huh7-shControl, Huh7-shGalectin-3, Huh7-shGalectin-3-vector and Huh7-shGalectin-3-β-catenin cells (bottom). Ctrl control, G3 Galectin-3. The results represent three independent experiments. *P < 0.05; **P < 0.01; ***P < 0.001.

Owing to its unique structure, Galectin-3 exhibits interactions with a plethora of ligands in both intracellular and extracellular compartments. Intracellular Galectin-3 regulates signalling pathways by interacting with cytoplasmic and nuclear proteins.^17^ Accumulating evidences suggested that β-catenin signalling was responsible for tumour angiogenesis and EMT induced by aberrant protein expression.^24–26^ Furthermore, the GSEA results indicated that Galectin-3 had a significantly positive association with the Wnt signalling pathway (Supplementary Fig. 6), and genes positively associated with Galectin-3 expression in this set were shown in Supplementary Table S1. Therefore, we wondered whether the modulation of angiogenesis and EMT by Galectin-3 depends on the activation of Wnt-β-catenin signalling in HCC cells. To explore this concept, we examined the expression of β-catenin in Galectin-3-overexpressing and knockdown cells. Interestingly, Galectin-3 did not affect the mRNA expression level of β-catenin (Supplementary Fig. 7a). Using Hep3B and Huh7 as representative cell lines, we detected the distribution of β-catenin in the whole cell, cytoplasm and nucleus. Intriguingly, a remarkably increased nuclear translocation of β-catenin was exhibited in Galectin-3-overexpressing cells, a significant decrease in which was seen in Galectin-3-knockdown cells (Supplementary Fig. 7b). IF staining clearly demonstrated that increased Galectin-3 expression was accompanied by the enhanced accumulation of β-catenin in the nucleus, whereas the inverse phenomenon was seen in Galectin-3-knockdown cells (Fig. 3a). These results indicate that Galectin-3 may modulate the nuclear translocation of β-catenin at the post-translational level.

It has been reported that inactivation of GSK-3β, one of the key components of the β-catenin degradation complex, via the PI3K-Akt signalling pathway causes β-catenin dephosphorylation and nuclear accumulation in colon cancer.^27^ Enrichment analysis (Fig. 2a) showed that Galectin-3 was significantly correlated with the PI3K-Akt signalling pathway; therefore, we explored whether PI3K-Akt signalling-mediated GSK-3β inactivation was involved in Galectin-3-induced β-catenin nuclear translocation in HCC cells. A high level of PI3K, Akt and GSK-3β phosphorylation was detected in Galectin-3-overexpressing cells, while a low level was seen in Galectin-3 knockdown cells (Fig. 3b). PI3K, Akt or GSK-3β inhibitors did not alter Galectin-3 expression (Fig. 3c). However, the PI3K inhibitor LY294002 significantly attenuated Akt and GSK-3β phosphorylation in Galectin-3-overexpressing cells; the Akt inhibitor AZD5363 effectively impaired Akt and GSK-3β phosphorylation with no effect on PI3K phosphorylation; and the GSK-3β inhibitor AR-A014418 suppressed GSK-3β phosphorylation but had no effect on PI3K or Akt phosphorylation (Fig. 3c). These results indicate that Galectin-3 enhanced GSK-3β phosphorylation via the activation of the PI3K-Akt signalling pathway. Moreover, PI3K, Akt and GSK-3β inhibitors effectively abolished the nuclear translocation of β-catenin induced by Galectin-3 (Fig. 3d). These data suggest that Galectin-3 activates the PI3K-Akt-GSK-3β signalling cascade, leading to increased β-catenin accumulation in the nucleus.

Subsequently, stable β-catenin knockdown was established in Galectin-3-overexpressing cells and stable β-catenin overexpression was established in Galectin-3-knockdown cells. We found that β-catenin knockdown partially reversed Galectin-3-induced angiogenesis and migration, both of which were significantly recovered following overexpression of β-catenin in Galectin-3-knockdown cells (Fig. 3e; Supplementary Fig. 8). The adhesion assay showed similar results in both Galectin-3-overexpressing and knockdown cells (Fig. 3f), indicating that β-catenin is crucial for Galectin-3-induced angiogenesis and EMT.

Next, we explored the target genes by which β-catenin signalling mediated Galectin-3-induced angiogenesis and EMT of HCC cells. A proteome angiogenesis array was performed in Galectin-3-overexpressing cells with or without stable β-catenin knockdown, and the results show that IGFBP3 was the cytokine whose levels were decreased the most following β-catenin knockdown (Fig. 3g; Supplementary Fig. 9). Accordingly, we performed a PCR array to identify the key participants of β-catenin signalling-mediated EMT in Galectin-3-overexpressing cells, and vimentin, NCAD and MMP3 displayed a significant reduction following β-catenin knockdown (Fig. 3h; Supplementary Fig. 10). To verify whether IGFBP3, vimentin, NCAD and MMP3 are directly regulated by the β-catenin/TCF4 transcription complex, we used the PROMO website to predict putative TCF4-binding sites in their promoter regions, followed by the performance of ChIP assays. By checking the canonical targets of β-catenin in the liver, including Axin2, Lgr5 and GS, we found that Galectin-3 indeed enhanced β-catenin-dependent transcription (Supplementary Fig. 11). As shown in Fig. 3i, TCF4 directly bound to the putative TCF4-binding site in the *IGFBP3 and VIM* promoter regions, and their expression showed an increase in Galectin-3 overexpression cells but a decrease in Galectin-3-knockdown cells, suggesting that β-catenin/TCF4 complex directly regulated *IGFBP3* and *VIM* transcription. Moreover, IF revealed that nuclear β-catenin was co-expressed with IGFBP3 and vimentin in HCC cells, which was dramatically increased in Galectin-3-overexpressing cells and decreased in knockdown cells (Fig. 3j); similar observations were obtained by western blotting (Fig. 3k). Taken together, these data indicate that β-catenin directly regulates IGFBP3 and vimentin expression, which are responsible for the Galectin-3-mediated increase in angiogenesis and EMT of HCC cells, respectively.

### Galectin-3 facilitates the tumorigenesis and lung metastasis of HCC cells in vivo via β-catenin signalling

**Fig. 4:**
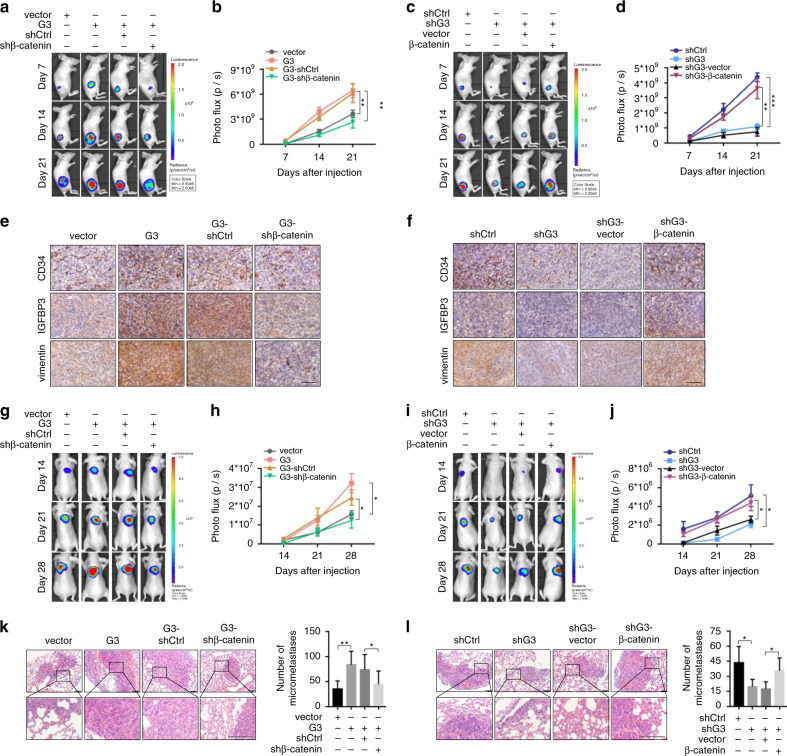
Galectin-3 facilitates tumorigenesis and lung metastasis of HCC cells in vivo via β-catenin signalling. **a**, **b** Representative images and photo flux of tumour growth of Hep3B-vector, Hep3B-Galectin-3, Hep3B-Galectin-3-shControl and Hep3B-Galectin-3-shβ-catenin cells in BALB/c nude mice at day 7, 14 and 21. **c**, **d** Representative images and photo flux of tumour growth of Huh7-shControl, Huh7-shGalectin-3, Huh7-shGalectin-3-vector and Huh7-shGalectin-3-β-catenin cells in BALB/c nude mice at day 7, 14 and 21. **e**, **f** IHC staining shows the expression of CD34, IGFBP3 and vimentin in xenografts. Scale bars, 100 μm. **g**, **h** Representative images and photo flux of lung metastasis of Hep3B-vector, Hep3B-Galectin-3, Hep3B-Galectin-3-shControl and Hep3B-Galectin-3-shβ-catenin cells in BALB/c nude mice at day 14, 21 and 28. **i**, **j** Representative images and photo flux of lung metastasis of Huh7-shControl, Huh7-shGalectin-3, Huh7-shGalectin-3-vector and Huh7-shGalectin3-β-catenin cells in BALB/c nude mice at day 14, 21 and 28. **k**, **l** H&E staining shows micro-metastatic pulmonary nodules of Hep3B-vector, Hep3B-Galectin-3, Hep3B-Galectin-3-shControl, Hep3B-Galectin-3-shβ-catenin, Huh7-shControl, Huh7-shGalectin-3, Huh7-shGalectin-3-vector and Huh7-shGalectin-3-β-catenin cells in BALB/c nude mice. Scale bars, 100 μm. Ctrl control, G3 Galectin-3. The results represent three independent experiments. *P < 0.05; **P < 0.01; ***P < 0.001.

To explore the effect of Galectin-3 on tumorigenesis and the role of β-catenin in this process in vivo, a BALB/c nude mouse xenograft model was constructed using Hep3B and Huh7 cells stably expressing luciferase. Mice with xenografts derived from Galectin-3-overexpressing cells displayed a higher photo flux (Fig. 4a, b) and tumour weight (Supplementary Fig. 12a), which was partially abrogated by β-catenin knockdown. In contrast, mice with xenografts derived from Galectin-3 knockdown cells exhibited a lower photo flux (Fig. 4c, d) and tumour weight (Supplementary Fig. 12b), which could be significantly restored by β-catenin overexpression. Consistent with the proposed regulation of angiogenesis and EMT by β-catenin, the expression patterns of CD34, IGFBP3 and vimentin in xenografts, as assessed by IHC staining, were similar to those seen in vitro (Fig. 4e, f; Supplementary Fig. 12c, d), supporting the protumour role of Galectin-3 in HCC tumorigenesis via β-catenin signalling.

Subsequently, we developed an in vivo lung metastasis model by injecting Hep3B and Huh7 cells stably expressing luciferase into the lateral tail vein of BALB/c nude mice. Galectin-3 overexpression contributed to a significant enhancement of metastatic growth in the lungs, as well as an increase in lung weight, which were partially reversed following β-catenin knockdown (Fig. 4g, h; Supplementary Fig. 12e). However, Galectin-3 knockdown reduced these parameters, which were reintroduced by overexpression of β-catenin (Fig. 4i, j; Supplementary Fig. 12f). Similar results were obtained regarding the number of micro-metastatic pulmonary nodules as assessed by H&E staining (Fig. 4k, l). Taken together, these results favour Galectin-3 as a promoter of HCC metastasis through β-catenin signalling.

### Blockade of Galectin-3-β-catenin signalling enhances the sensitivity of HCC cells to sorafenib

**Fig. 5:**
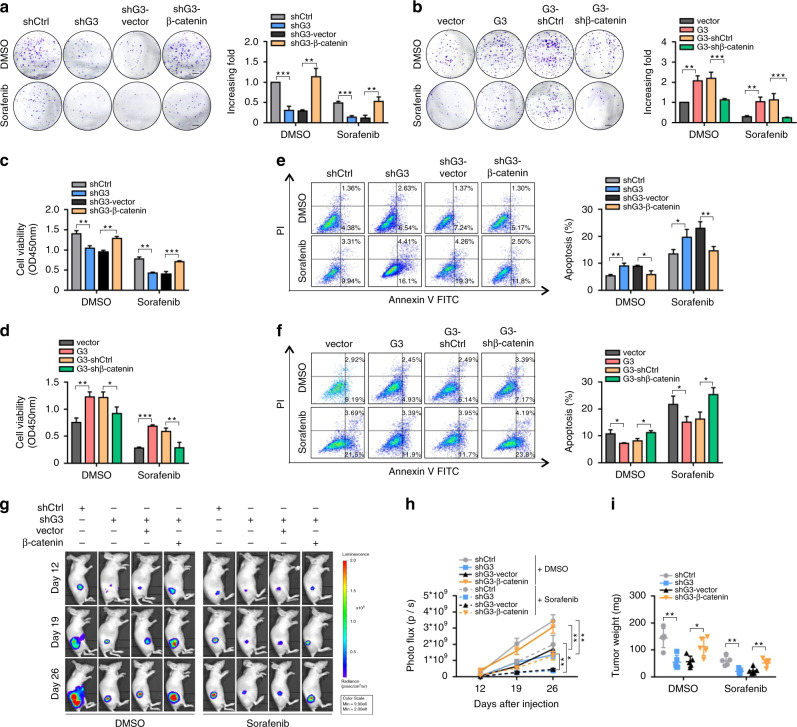
Molecular deletion of Galectin-3-β-catenin signalling synergistically improves the antitumour effect of sorafenib in vitro and in vivo. **a**–**f** After cell adhesion, DMSO or sorafenib (20 μmol/mL) were administered every two days for the colony formation assay, (**a, b**), CCK8 assay (**c, d**) and apoptosis assay (**e, f**) in Hep3B-vector, Hep3B-Galectin-3, Hep3B-Galectin-3-shControl, Hep3B-Galectin-3-shβ-catenin, Huh7-shControl, Huh7-shGalectin-3, Huh7-shGalectin-3-vector and Huh7-shGalectin-3-β-catenin cells. Scale bars in **a** and **b**, 5 mm. **g, h** Representative images and photo flux of tumour growth of Hep3B-vector, Hep3B-Galectin-3, Hep3B-Galectin-3-shControl, Hep3B-Galectin-3-shβ-catenin, Huh7-shControl, Huh7-shGalectin-3 Huh7-shGalectin-3-vector and Huh7-shGalectin-3-β-catenin cells in BALB/c nude mice treated with DMSO or sorafenib at day 12, 19, and 26. **i** Mice were sacrificed at day 27. The tumour weights were measured. Ctrl control, G3 Galectin-3. The results represent three independent experiments. *P < 0.05; **P < 0.01; ***P < 0.001.

Sorafenib, a small-molecule antitumour drug that acts by inhibiting angiogenesis and tumour growth, is widely used in unresectable or metastatic HCC.^3,4^ We found that Galectin-3 knockdown remarkably enhanced the inhibitory effect of sorafenib on HCC cell (Huh7) proliferation in the colony formation and CCK8 assays, which was disrupted by β-catenin overexpression (Fig. 5a, c). Opposing results were seen in Galectin-3-overexpressing Hep3B cells with or without β-catenin knockdown (Fig. 5b, d). Furthermore, Galectin-3 or β-catenin knockdown synergistically enhanced the sensitivity of HCC cells to sorafenib in cell apoptosis assay, whereas reduced sensitivity of HCC cells to sorafenib was observed in Galectin-3- or β-catenin-overexpressed cells (Fig. 5e, f). To confirm whether Galectin-3-β-catenin signalling blockade and sorafenib administration had a synergistic effect on tumour growth in vivo, Huh7 cells stably expressing luciferase were injected subcutaneously into BALB/c nude mice, which were then treated orally with sorafenib. We observed that knockdown of Galectin-3 synergistically enhanced the antitumour effect of sorafenib as compared with either Galectin-3 knockdown or sorafenib alone, while β-catenin overexpression abrogated this synergistic effect (Fig. 5g−i). In addition, we also found that Galectin-3 and β-catenin knockdown has an additive effect on the sensitivity of HCC cells to sorafenib in the in vitro experiments (Supplementary Fig. 13). Therefore, blockade of Galectin-3-β-catenin signalling may serve as a potential adjuvant therapeutic strategy in HCC patients treated with sorafenib.

### Galectin-3-β-catenin-IGFBP3/vimentin axis is correlated with vascular invasion-mediated sorafenib resistance and presents a strong correlation in large-scale clinical samples

**Fig. 6:**
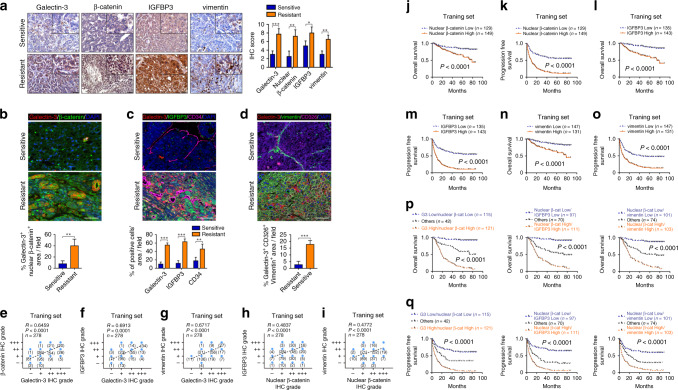
High-level Galectin-3-β-catenin-IGFBP3/vimentin axis is correlated with vascular invasion-mediated sorafenib resistance and poor prognosis in large-scale clinical samples. **a**–**d** Sorafenib-sensitive patients without vascular invasion and sorafenib-resistant patients with vascular invasion were selected from the training set. **a** IHC staining shows the expression levels of Galectin-3, β-catenin, IGFBP3 and vimentin. Scale bars, 50 μm. **b**–**d** Tissue IF staining shows the co-expression pattern of Galectin-3 (red) and β-catenin (green) (**b**); Galectin-3 (red), IGFBP3 (green) and CD34 (magenta) (**c**); and Galectin-3 (red), vimentin (green) and CD326 (magenta) (**d**). DAPI, blue. Figure panel pairs the representative images taken with different zooming options; Scale bars, 100 μm. **e**–**h** Spearman’s correlation analysis for the relationship between Galectin-3 and nuclear β-catenin (**e**); Galectin-3 and IGFBP3 (**f**); Galectin-3 and vimentin (**g**); nuclear β-catenin and IGFBP3 (**h**) and nuclear β-catenin and vimentin (**i**) in the training set according to IHC staining. **j**–**o** Analysis of the differences between the high and low expression of nuclear β-catenin (**j, k**), IGFBP3 (**l, m**) and vimentin (**n, o**) for the OS and PFS based on IHC staining in the training set. **p, q** Kaplan–Meier analysis of the OS (**p**) and PFS (**q**) in the training set containing 278 HCC patients according to both Galectin-3 and nuclear β-catenin expression (left), nuclear β-catenin and IGFBP3 expression (middle) and nuclear β-catenin and vimentin expression (right), as assessed by IHC staining. No without vascular invasion, Yes with vascular invasion. β-cat β-catenin, G3 Galectin-3. The results represent three independent experiments. *P < 0.05; **P < 0.01; ***P < 0.001.

Next, we investigated whether the expression levels of Galectin-3-β-catenin-IGFBP3/vimentin axis were involved in sorafenib resistance in clinical samples. Sorafenib-sensitive patients without vascular invasion and sorafenib-resistant patients with vascular invasion were selected from the training set. We firstly detected the expression of Galectin-3-β-catenin-IGFBP3/vimentin axis by IHC staining. As shown in Fig. 6a, the expression levels of Galectin-3, nuclear β-catenin, IGFBP3 and vimentin in sorafenib-resistant patients were markedly higher than those in sorafenib-sensitive patients. In IF colocalisation staining, nuclear β-catenin displayed a simultaneously low and high expression pattern with Galectin-3 in sorafenib-sensitive and -resistant patients, respectively (Fig. 6b). Additionally, we found that IGFBP3 and CD34 were simultaneously downregulated and upregulated with Galectin-3 in sorafenib-sensitive and -resistant tissues, respectively (Fig. 6c), and the expression pattern of vimentin and CD326 was similar to that of Galectin-3 (Fig. 6d). Subsequently, the correlation of Galectin-3-β-catenin-IGFBP3/vimentin axis was explored by IHC staining analysis in large-scale samples from the training, testing and vascular invasion sets. A significantly positive correlation was observed between Galectin-3 and nuclear β-catenin expression (Fig. 6e; Supplementary Fig. 14a). Furthermore, both IHC staining and TCGA data for HCC demonstrate that the expression of Galectin-3 had a close positive correlation with that of IGFBP3 and vimentin in a large proportion of HCC samples (Fig. 6f, g; Supplementary Fig. 14b−d). In addition, the expression level of nuclear β-catenin also showed a positive association with that of IGFBP3 and vimentin in all three HCC sets (Fig. 6h, i; Supplementary Fig. 14e, f), suggesting the probably direct regulation of β-catenin on IGFBP3 and vimentin in vivo. Collectively, these data indicate that the Galectin-3-β-catenin-IGFBP3/vimentin axis are closely correlated in HCC patients, and high expression levels are associated with vascular invasion-mediated sorafenib resistance.

### High-level β-catenin-IGFBP3/vimentin signalling predicts poor prognosis and vascular invasion in patients with HCC

Based on the above results, we next assessed whether the expression level of β-catenin-IGFBP3/vimentin signalling has a relationship with poor prognosis in patients with HCC. Kaplan–Meier survival analysis based on IHC staining indicated that patients with high-level nuclear β-catenin, IGFBP3, or vimentin had a shorter OS and PFS no matter in training (Fig. 6j-o), testing or vascular invasion set (Supplementary Fig. 15a-f). Moreover, we further stratified the 278 HCC tissues from training set into three groups according to the expression levels of Galectin-3 and nuclear β-catenin, nuclear β-catenin and IGFBP3 and nuclear β-catenin and vimentin. We found that patients in groups with high expression of both Galectin-3 and nuclear β-catenin, both nuclear β-catenin and IGFBP3 and both nuclear β-catenin and vimentin presented the significantly worst OS (Fig. 6p) and PFS rates (Fig. 6q).

We further conducted ROC analyses in all the three sets to assess the discriminatory ability of β-catenin-IGFBP3/vimentin signalling. As shown in Supplementary Fig. 16a–f, the AUCs of nuclear β-catenin, IGFBP3 and vimentin were all more than 0.75 in the three sets in the assessment of OS and PFS. Meanwhile, nuclear β-catenin, IGFBP3 and vimentin also performed well in assessment of vascular invasion with all AUCs above 0.7 (Supplementary Fig. 16g-i). These results suggested that the β-catenin-IGFBP3/vimentin signalling had good sensitivity and specificity to predict survival and vascular invasion in patients with HCC. Hence, Galectin-3-β-catenin signalling might be the potential biomarker for predicating vascular metastasis and poor survival in HCC patients.

## Discussion

Despite recent advances in diagnostics and therapeutics, the outcome for HCC patients remains poor and distant metastasis represents a major barrier to successful treatment in most patients.^1,2,28,29^ Mechanistically, we revealed that Galectin-3 modulated angiogenesis- and EMT-driven tumour metastasis via activation of the PI3K-Akt-GSK-3β-β-catenin signalling cascade by targeting IGFBP3 and vimentin in the HCC tumour microenvironment (Supplementary Fig. 17). Clinically, Galectin-3 expression showed a strong correlation with β-catenin-IGFBP3/vimentin signalling in HCC samples, a high level of which was associated with vascular metastasis-driven sorafenib resistance and a poor prognosis in patients with HCC. Our study may serve as a rationale for targeting the HCC-intrinsic Galectin-3-β-catenin signalling in a novel therapeutic application to treat metastatic or sorafenib-resistant patients.

As a pleiotropic protein, Galectin-3 has been reported to be extensively implicated in the regulation of multiple cell processes during tumour metastasis in several types of cancer.^30–32^ In the present study, we found that Galectin-3 induced HCC metastasis by regulating EMT and angiogenesis through activation of PI3K-Akt-GSK-3β signalling. However, downregulation of Gelectin-3 inversely was also found in breast cancer, prostate cancer and endometrial cancer tissues,^33–36^ which might be due to changes in its cytosolic and nuclear expression pattern, activating distinct molecular mechanisms in different cancer types.^37^ In addition, Galectin-3 was generally considered as a cell adhesion molecule, but we found that Galectin-3 overexpression suppressed HCC cell adhesion whilst Galectin-3 suppression enhanced cell adhesion in this study. According to the in-depth study, we speculated that the reduced effect of Galectin-3-induced EMT on cell adhesion might exceed the enhanced effect of Galectin-3 itself as an adhesion molecule to cell adhesion in Galectin-3 overexpression cells, eventually leading to the reduction of cell adhesion. Also, our speculation indirectly supported the phenomenon of Galectin-3 promoting tumour cell metastasis in our study as well as other literatures.^38,39^

Interestingly, we observed a relationship between Galectin-3 and cell cytokine production in the GO functional analysis in the study. Actually, existing literatures have extensively implicated Galectin-3 as master regulators of cytokine production. In 2002, Galectin-3 has been identified as an amplifier of the inflammatory cascade by interfering with cytokine secretion, influencing tumour progression and metastasis.^40^ Partridge et al. revealed that Galectin-3 delayed the removal of multiple cytokines by constitutive endocytosis through cross-link to Mgat5-modified N-glycans and thereby promoted cytokine-mediated leukocyte signalling, phagocytosis, and extravasation in vivo.^41^ Chen et al. reported that increased levels of Galectin-3 in patients with colorectal cancer induces secretion of several metastasis-promoting cytokines from the blood vascular endothelium, including IL-6, G-CSF, sICAM-1 and granulocyte macrophage colony-stimulating factor.^42^ In pancreatic ductal adenocarcinoma, Galectin-3 could also activate pancreatic stellate cells to produce inflammatory cytokines, such as IL8, GMCSF, CXCL1 and CCL2, facilitating the interactions between tumour cell and stroma.^43^

It has been reported that β-catenin signalling is also constitutively activated through activating mutations of its gene, CTNNB1. To discriminate the effects of Galectin-3 cascade versus CTNNB1 mutations in the activation of β-catenin pathway, we also assessed the mutational status of β-catenin in in exon 3. Consistent with other literature^44^ and TCGA data (mutation rate: 28.8% in grade I, 23.4% in grade II–IV), we also found that this mutation pattern mainly existed in well-differentiated HCC in this study (mutation rate: 27.9% in grade I, 8.1% in grade II–III). Moreover, the nuclear accumulation of β-catenin in patients with CTNNB1 mutation was higher than those without mutations according to IHC staining (Supplementary Fig. 18a). However, the expression level of Galectin-3 was relatively low in patients with CNTTB1 mutation (Supplementary Fig. 18b), which was also observed in TCGA dataset of HCC (Supplementary Fig. 18c). This inverse pattern between CTNNB1 mutation and Galectin-3 indicates that they do not activate β-catenin in a synergistic manner, but in an independent manner. And we think that the effect of Galectin-3 cascade was dominant in activation of β-catenin pathway in this study.

In summary, we show that Galectin-3 modulated angiogenesis- and EMT-driven metastasis via activation of the β-catenin signalling cascade by targeting IGFBP3 and vimentin in the HCC tumour microenvironment. Clinically, high expression levels of the components of the Galectin-3-β-catenin-IGFBP3/vimentin axis were closely correlated with vascular metastasis-driven sorafenib resistance and poor survival in large-scale HCC samples. The findings are of great significance for the further understanding of the mechanism underlying HCC metastasis. The Galectin-3-β-catenin axis may serve as a biomarker for predicting vascular invasion and poor prognosis, in addition to providing a set of effective targets for anti-metastatic HCC therapies. However, the clinical application of this axis requires further investigation.

## Supplementary information

Supplementary file

## Data Availability

All data generated or analysed during this study are included in this published article [and its supplementary information files].
