# Supplementary material for: Galectin-3 favours tumour metastasis via the activation of β-catenin signalling in hepatocellular carcinoma
Source: Br J Cancer. 2020 Aug 17;123(10):1521–34. doi: 10.1038/s41416-020-1022-4 (PMC7653936; doi:10.1038/s41416-020-1022-4)
Supplement: Supplementary file 1 — Supplementary file [file 41416_2020_1022_MOESM1_ESM.docx]

**Supplementary Figures and legends**


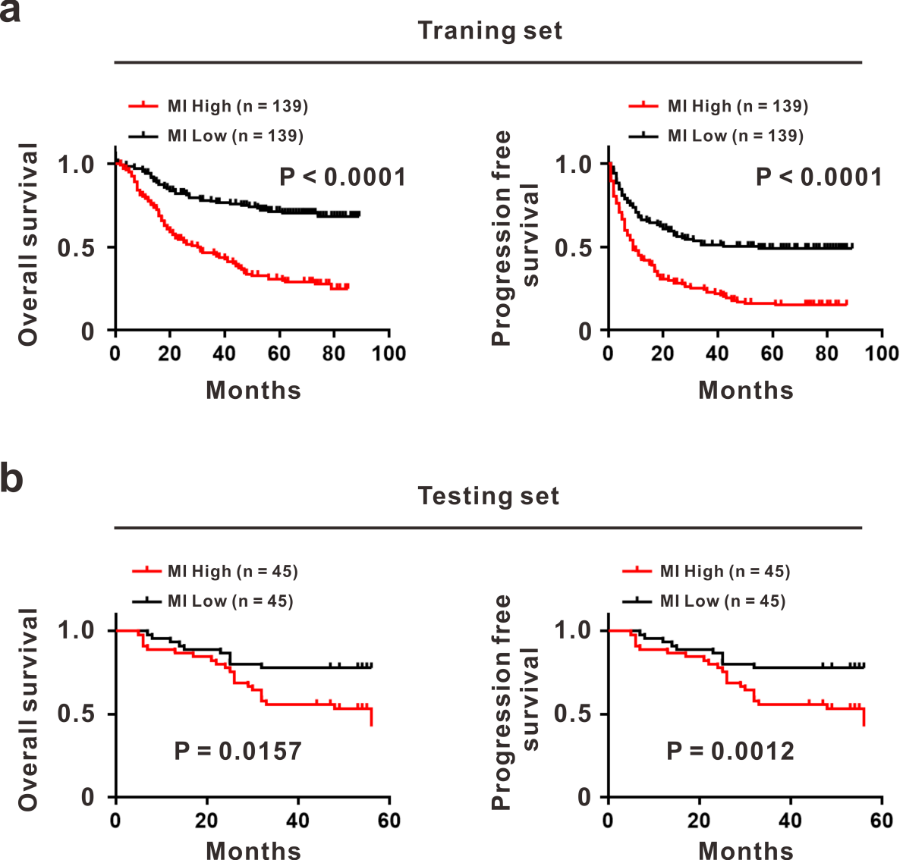


**Supplementary Fig. 1. High-level mitotic index (MI) was closely associated with poor overall survival and progression-free rates.** Survival analyses between “MI high” and “MI low” groups for the overall survival (left) and progression-free survival (right) based on HE staining in training **(a)** and testing **(b)** sets.


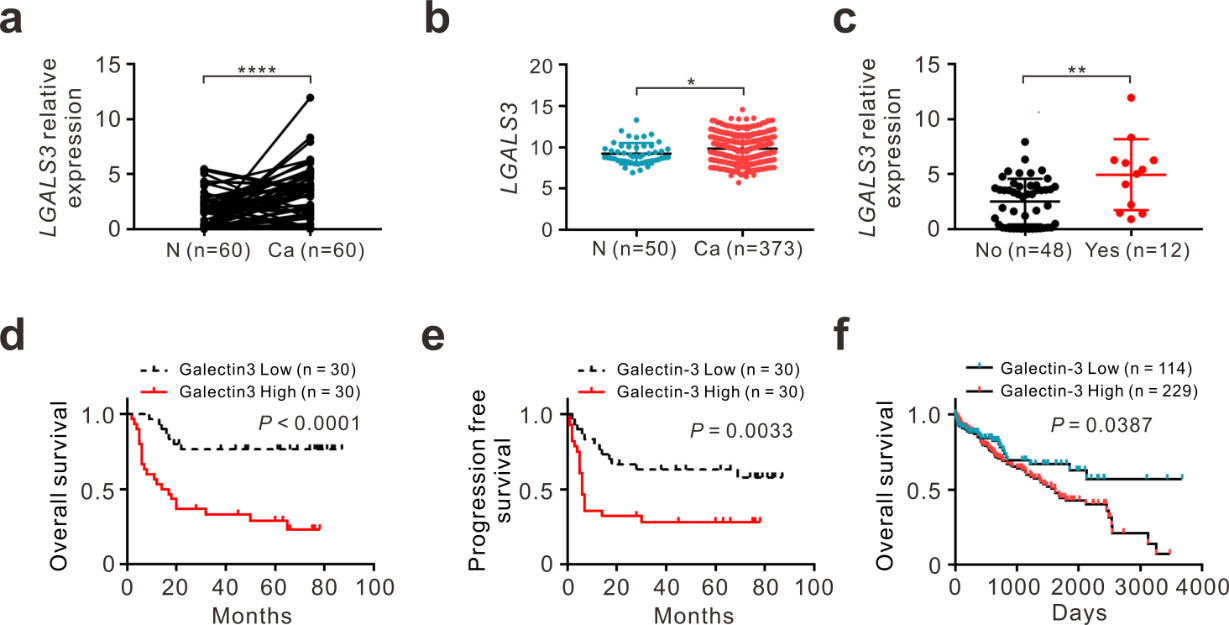


**Supplementary Fig. 2. Galectin-3 was upregulated in HCC at mRNA level and correlated with poor prognosis.** **(a)** qPCR detected the mRNA expression of *LGALS3* in 60 HCC tissues and paired adjacent normal tissues. **(b)** The expression level of *LGALS3* in RNA-sequencing data form TCGA including 50 normal liver tissues and 373HCC tissues. **(c)** The mRNA expression of *LGALS3* in HCC tissues with or without vascular invasion based on qPCR. **(d, e)** Survival analyses between “Galectin-3 high (n = 30)” and “Galectin-3 low (n = 30)” groups for the overall survival **(e)** and progression-free survival **(e)** based on qPCR in 60 clinical samples of HCC. **(f)** Survival analyses between “Galectin-3 high (n = 229)” and “Galectin-3 low (n = 114)” groups for the overall survival in the TCGA database of HCC. N, normal; Ca, cancer. The results were represented of three independent experiments. **P* < 0.05; ***P* < 0.01; *****P* < 0.0001.


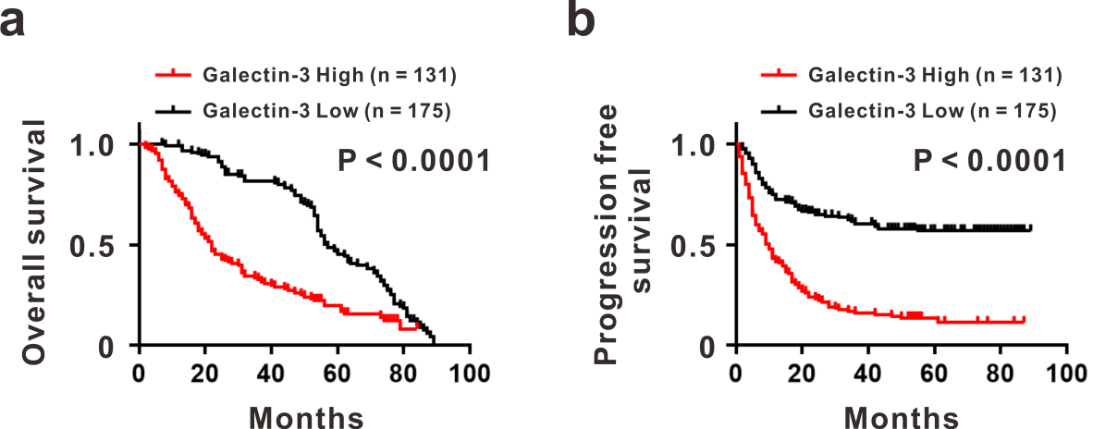


**Supplementary Fig. 3. High-level Galectin-3 expression is closely associated with poor prognosis in non-vascular invasion set.** Survival analyses of the differences between the high or low expression of Galectin-3 for the overall survival **(a)** and progression-free survival **(b)** based on IHC staining in non-vascular invasion set.


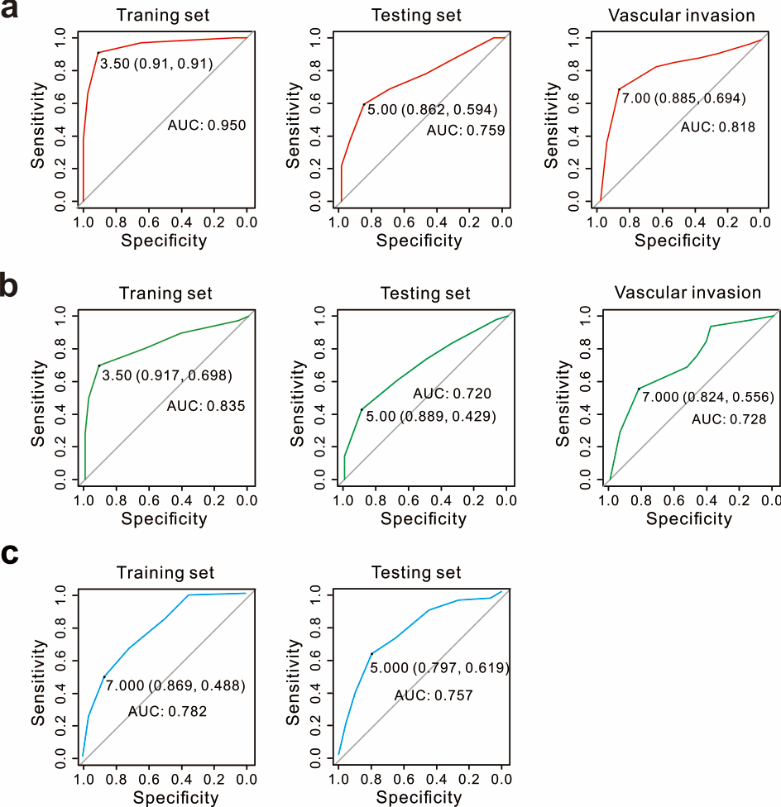


**Supplementary Fig. 4. ROC curves and corresponding AUCs of Galectin-3 for OS (a), PFS (b) in training (left), testing (middle) and vascular invasion (right) sets, as well as AUCs for vascular invasion in training (left) and testing (right) sets (c).**


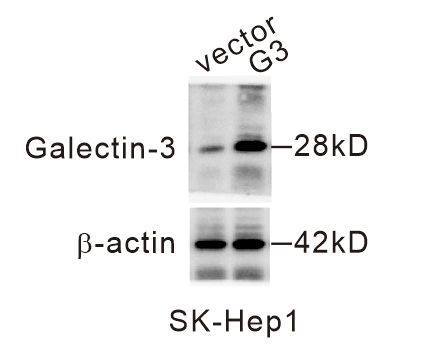


**Supplementary Fig. 5. The raw data for the SK-Hep1 blot in figure 2f.**


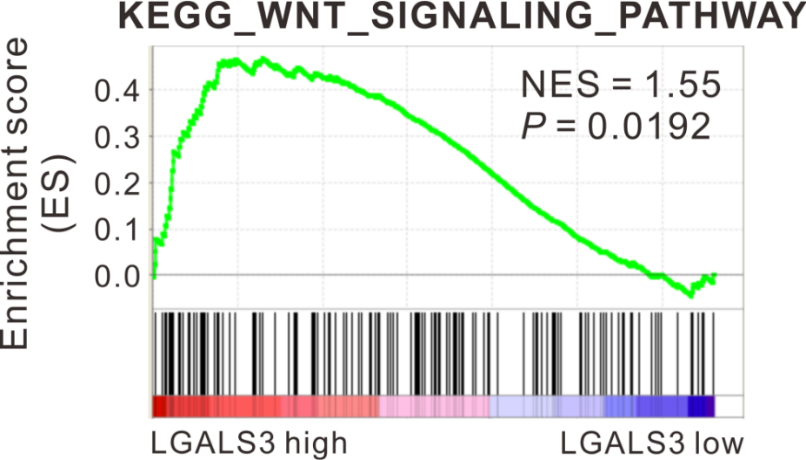


**Supplementary Fig. 6. GSEA based on the mRNA sequencing data from TCGA of HCC to analyze the correlation of Galectin-3 with Wnt signaling pathway.**


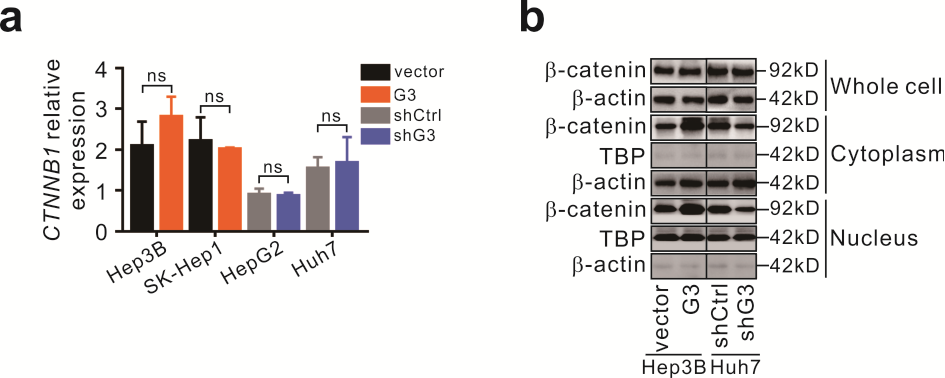


**Supplementary Fig. 7. (a)** qPCR analyzed the mRNA expression of *CTNNB1* in Hep3B-vector, Hep3B-Galectin-3, SK-Hep1-vector, SK-Hep1-Galectin-3, HepG2-shControl, HepG2-shGalectin-3, Huh7-shControl, and Huh7-shGalectin-3 cells. **(b)** Western blot analyzed the expression of β-catenin in the whole cell, cytoplasm, and nucleus of Hep3B-vector, Hep3B-Galectin-3, Huh7-shControl, and Huh7-shGalectin-3 cells. β-actin and TBP served as the endogenous control of cytoplasm and nucleus, respectively. Ctrl, Control; G3, Galectin-3. The results were represented of three independent experiments.


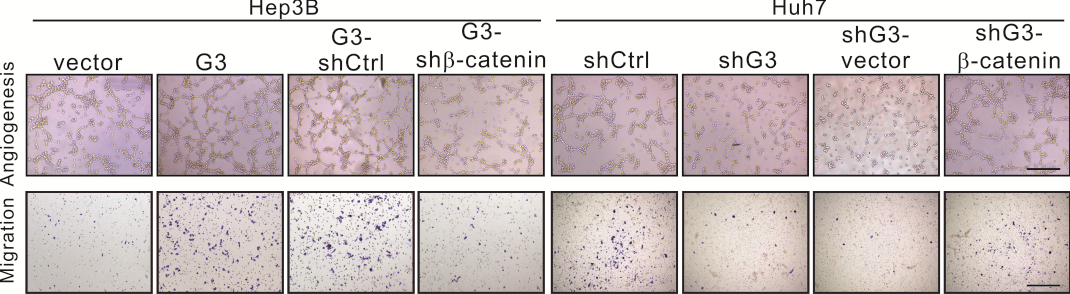


**Supplementary Fig. 8. Representive images of tube formation assay and transwell migration assay. Scale bars, 200μm.**


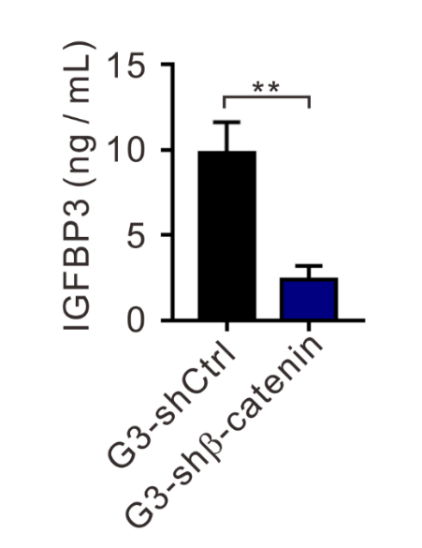


**Supplementary Fig. 9. ELISA detected the expression of IGFBP3 in the supernatants collected from shControl or shβ-catenin of Galectin-3-overexpressed cells.** The results were represented of three independent experiments. Ctrl, Control; G3, Galectin-3. ***P* < 0.01.


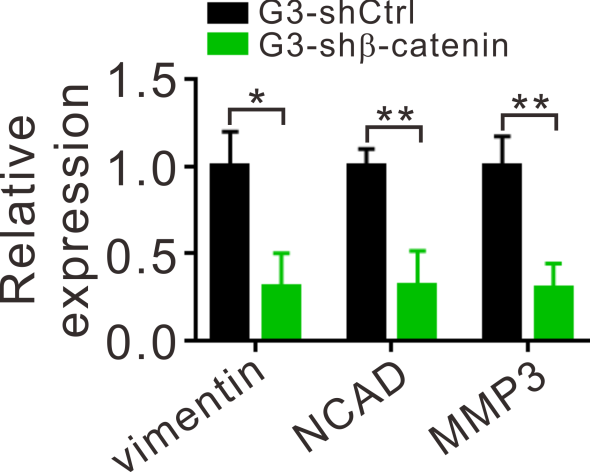


**Supplementary Fig. 10.** qPCR analysis for the expression of vimentin, NCAD, and MMP3 in shControl cells shβ-catenin Hep3B cells based on Galectin-3 overexpression. The results were represented of three independent experiments. Ctrl, Control; G3, Galectin-3. **P* < 0.05, ***P* < 0.01.


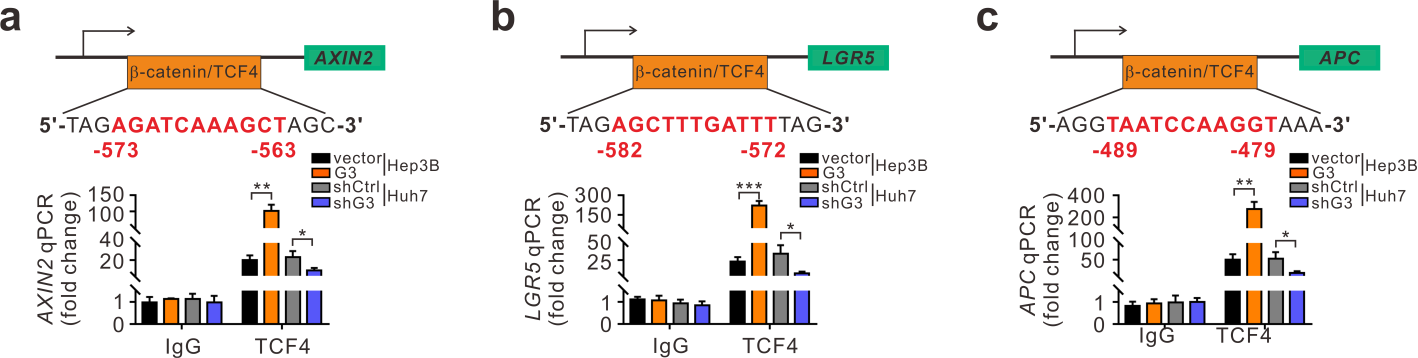


**Supplementary Fig. 11.** Analysis of the *AXIN2* **(a)**, *LGR5* **(b)**, and *VIM* **(c)** promoter identified a TCF4 binding site. CHIP was performed using IgG and TCF4 antibodies, followed by qPCR in Hep3B-vector, Hep3B-Galectin-3, Huh7-shControl, and Huh7-shGalectin-3 cells. The results were represented of three independent experiments. Ctrl, Control; G3, Galectin-3. **P* < 0.05, ***P* < 0.01, ****P* < 0.001.


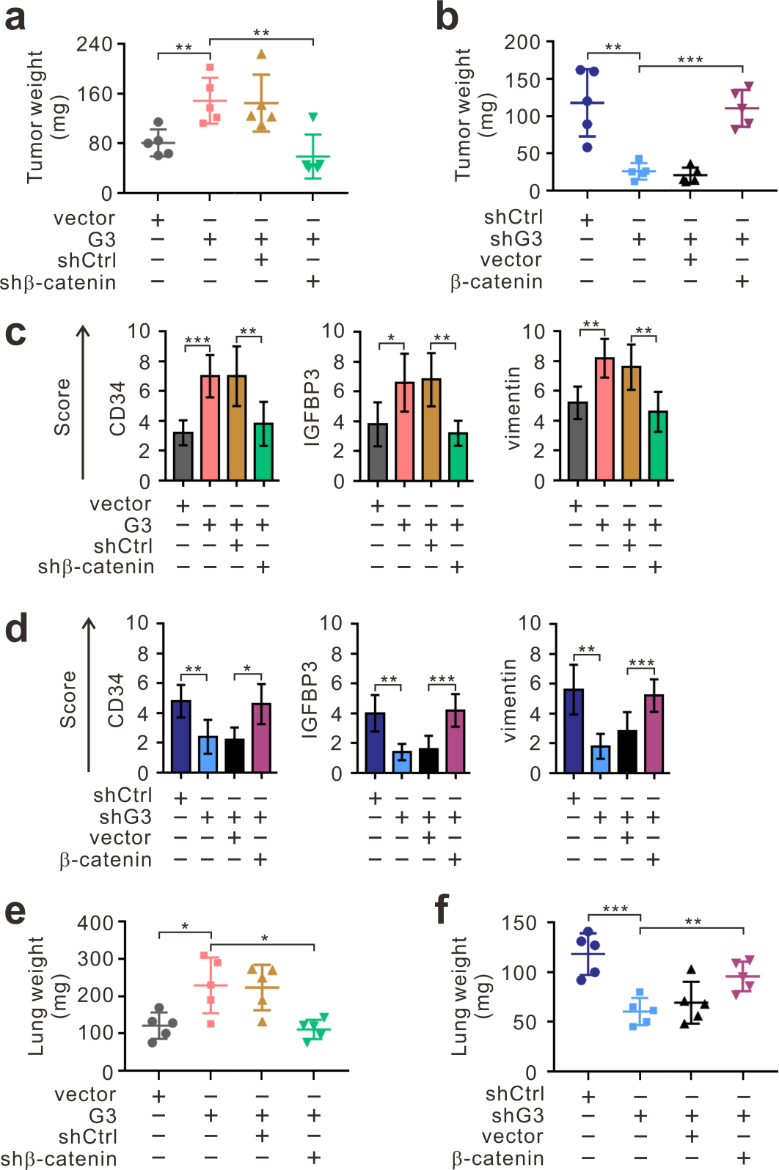


**Supplementary Fig. 12. Galectin-3 facilitates the tumorigenesis and lung metastasis of HCC cells *in vivo* via β-catenin signaling.** **(a)** Mice were sacrificed at day 22. The tumor weight was measured in Hep3B-vector, Hep3B-Galectin-3, Hep3B-Galectin-3-shControl, Hep3B-Galectin-3-shβ-catenin groups. **(b)** Mice were sacrificed at day 22. The tumor weight was measured xenografts from in Huh7-shControl, Huh7-shGalectin-3 Huh7-shGalectin-3-vector, and Huh7-shGalectin-3-β-catenin groups. **(c, d)** Statistical graphs of IHC staining for the expression of CD34, IGFBP3, and vimentin in xenografts. **(e)** Mice were sacrificed at day 29. The lung weight was measured in Hep3B-vector, Hep3B-Galectin-3, Hep3B-Galectin-3-shControl, Hep3B-Galectin-3-shβ-catenin groups. **(f)** Mice were sacrificed at day 29. The lung weight was measured in Huh7-shControl, Huh7-shGalectin-3 Huh7-shGalectin-3-vector, and Huh7-shGalectin-3-β-catenin groups. The results were represented of three independent experiments. Ctrl, Control; G3, Galectin-3. **P* < 0.05, ***P* < 0.01, ****P* < 0.001.


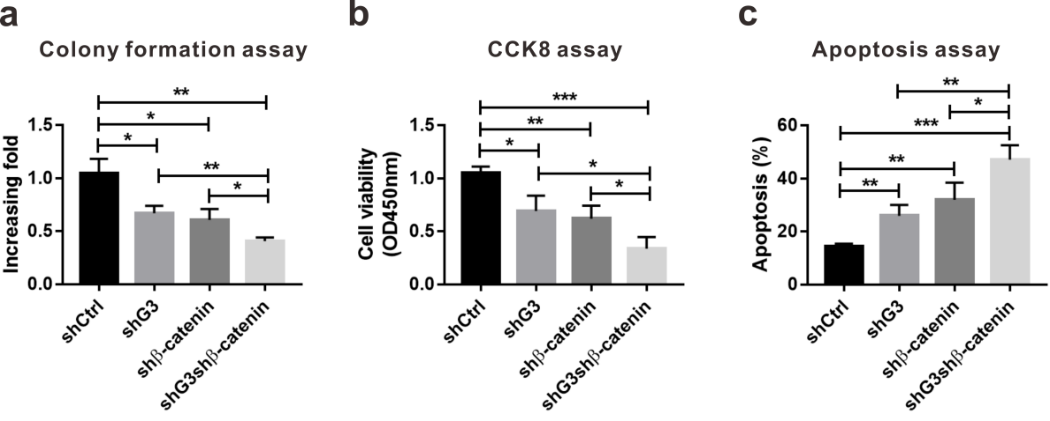


**Supplementary Fig. 13. Galectin-3 and β-catenin knockdown has an additive effect on the sensitivity of HCC cells to sorafenib.** After cell adhesion, sorafenib (20 μmol/ml) were administrated every two days for colony formation assay **(a)**, CCK8 assay **(b)**, and apoptosis assay using flow cytometry **(c)** in Huh7-shCtrl, Huh7-shG3, Huh7-shβ-catenin, Huh7-shG3shβ-catenin cells. The results were represented of three independent experiments. Ctrl, Control; G3, Galectin-3. **P* < 0.05, ***P* < 0.01, ****P* < 0.001.


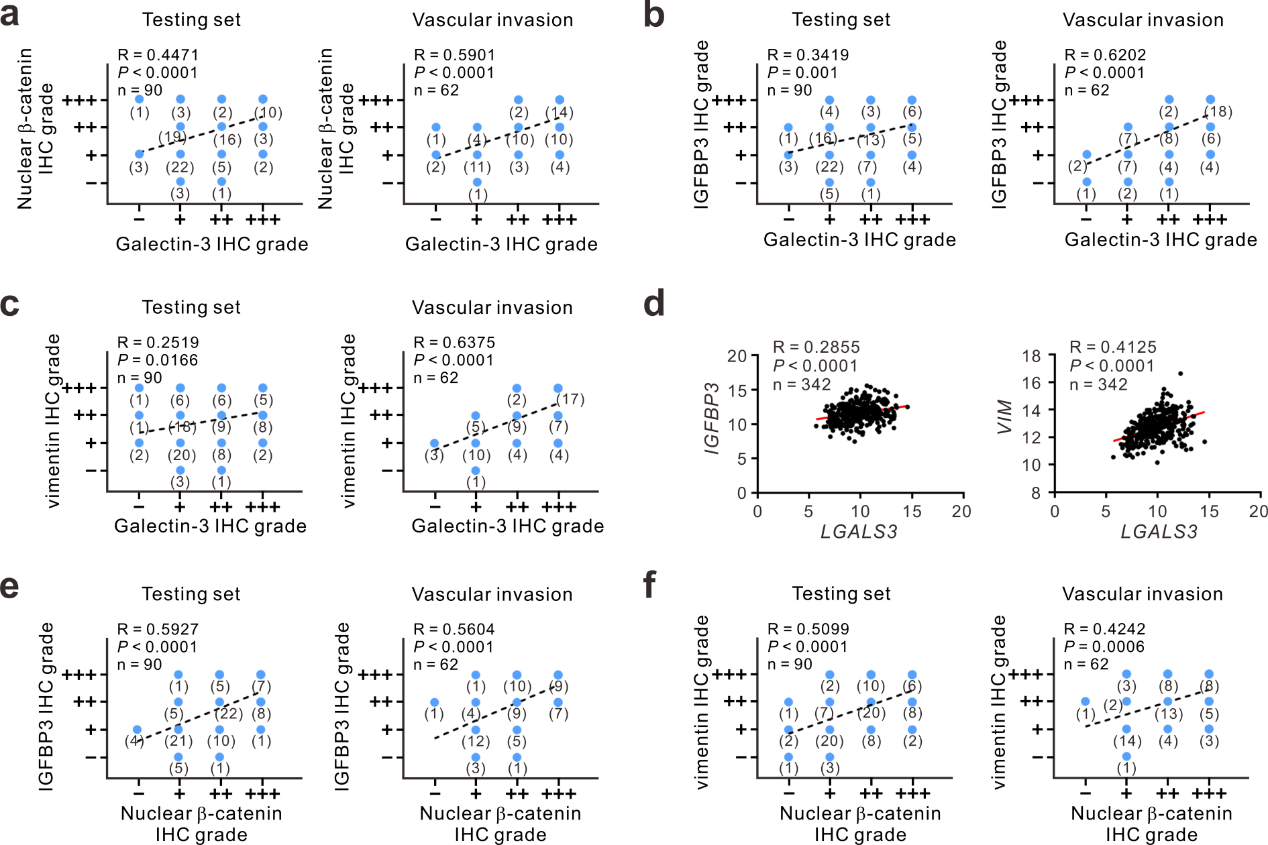


**Supplementary Fig. 14.** **Galectin-3-β-catenin-IGFBP3/vimentin axis presents a strong correlation in large-scale clinical samples. (a-c, e-f)** Spearman’s correlation analyses for the correlation between Galectin-3 and nuclear β-catenin **(a)**, Galectin-3 and IGFBP3 **(b)**, Galectin-3 and vimentin **(c)**, nuclear β-catenin and IGFBP3 **(f)**, nuclear β-catenin and vimentin **(f)** in testing (left) and vascular invasion (right) sets according to IHC staining results. **(d)** Spearman’s correlation analysis was performed to assess the correlation of *LGALS3* with *IGFBP3* (left) and *VIM* (right) in the TCGA database of HCC


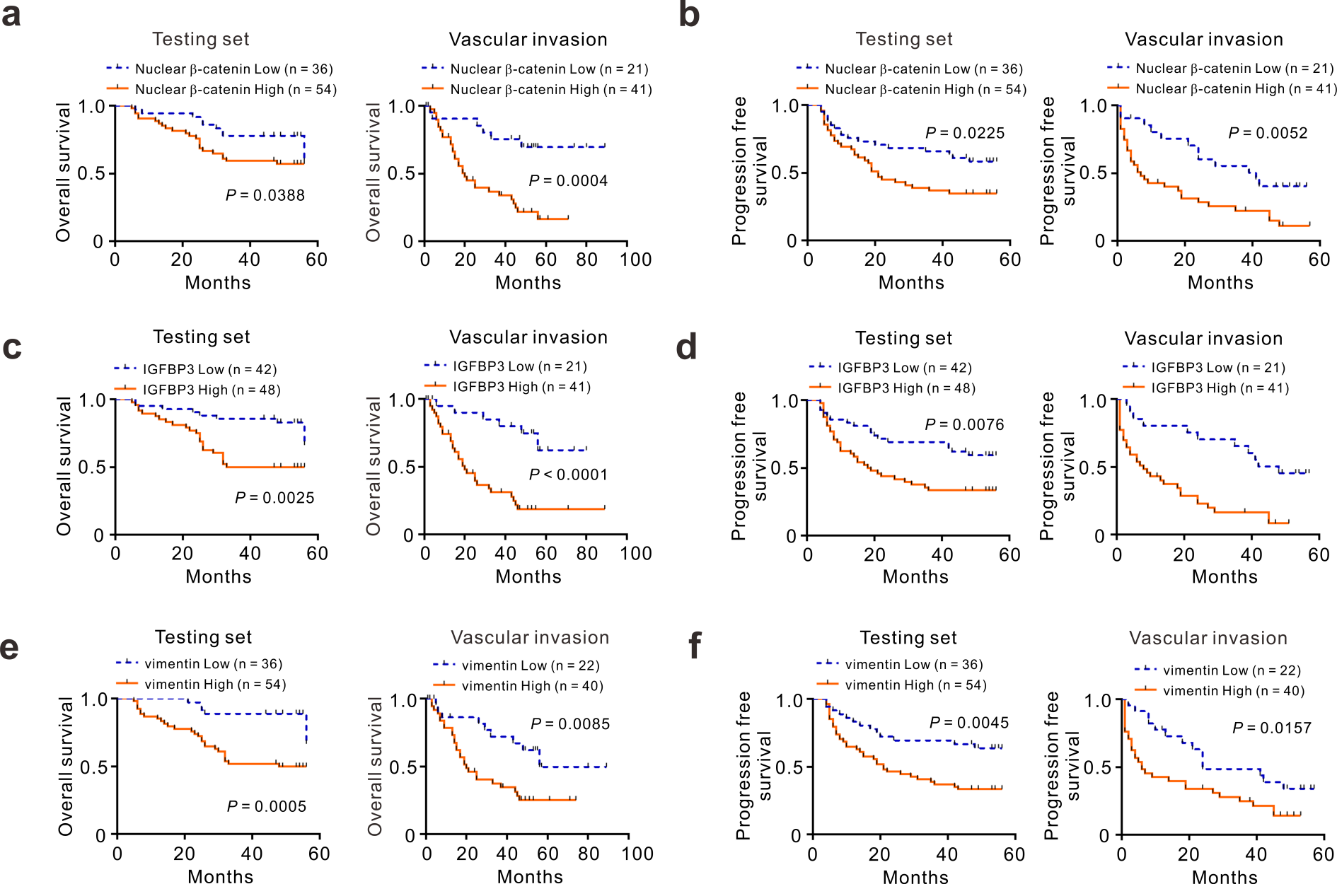


**Supplementary Fig. 15. High-level β-catenin-IGFBP3/vimentin expression is closely associated with vascular metastasis and poor prognosis in patients with HCC.** Survival analyses of the differences between the high or low expression of nuclear β-catenin **(a, b)**, IGFBP3 **(c, d)**, and vimentin **(e, f)** for the overall survival and progression-free survival based on IHC staining in testing (left) and vascular invasion (right) sets.


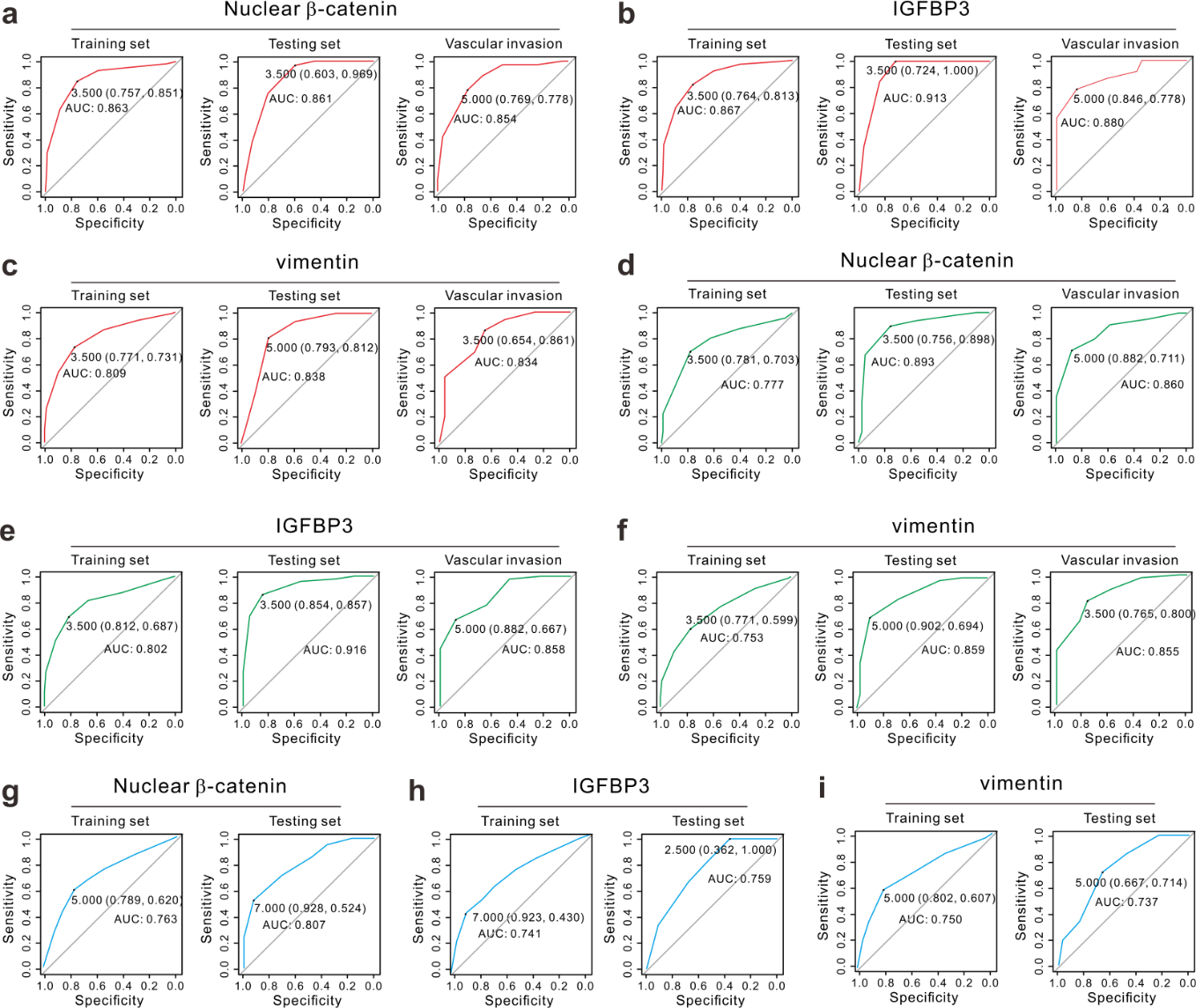


**Supplementary Fig. 16. ROC analyses for the β-catenin-IGFBP3/vimentin signaling in large scale patients with HCC.** Based on the IHC staining results of nuclear β-catenin, IGFBP3, and vimentin, ROC analysis was performed. **(a-c)** ROC curves of nuclear β-catenin **(a)**, IGFBP3 **(b)**, and vimentin **(c)** for OS in training (left), testing (middle) and vascular invasion (right) sets. **(d-f)** ROC curves of nuclear β-catenin **(d)**, IGFBP3 **(e)**, and vimentin **(f)** for PFS in training (left), testing (middle) and vascular invasion (right) sets. **(g-i)** ROC curves of nuclear β-catenin **(g)**, IGFBP3 **(h)**, and vimentin **(i)** for vascular invasion in training (left) and testing (right) sets. The results were represented of three independent experiments.


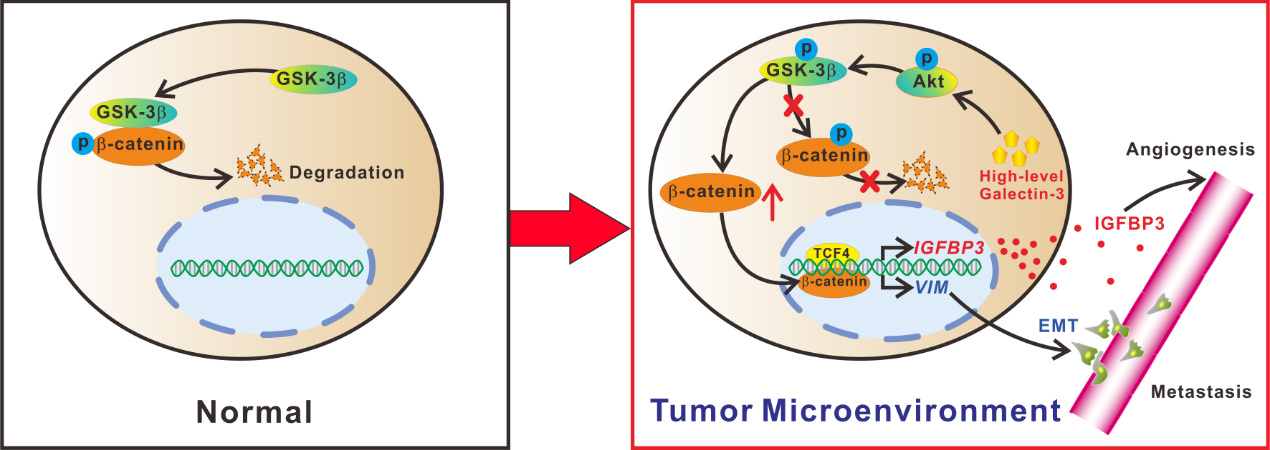


**Supplementary Fig. 17. Schematic diagram.**


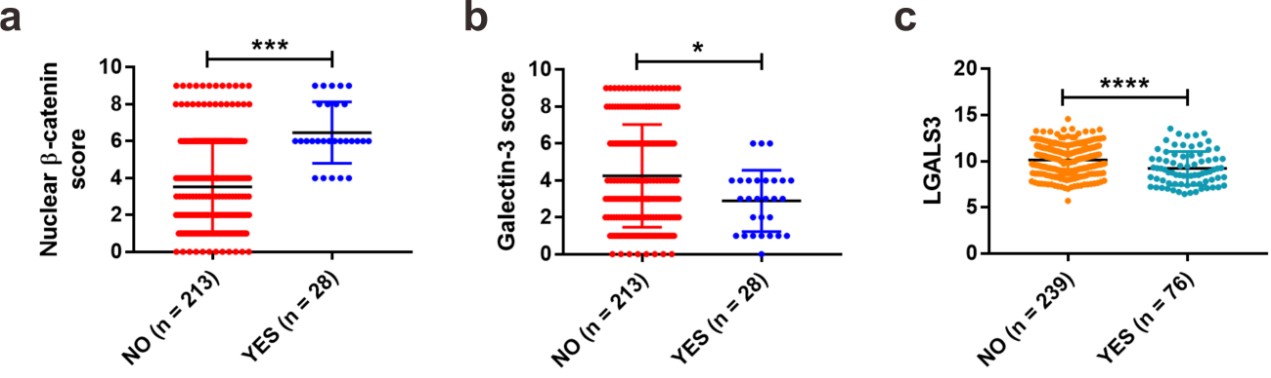


**Supplementary Fig. 18.** Inverse pattern between CTNNB1 mutation and Galectin-3 in HCC. **(a, b)** IHC scores of nuclear b-catenin and Galectin-3 in patients with or without CTNNB1 mutations in the training set. **(c)** LGALS3 expression in patients with or without CTNNB1 mutations in the TCGA dataset of HCC. YES, with CTNNB1 mutations; NO, without CTNNB1 mutations. **P* < 0.05, ****P* < 0.001, *****P* < 0.0001.

**Supplementary Table S1. Genes positively associated with Galectin-3 expression in the set of Wnt signaling pathway.**

| **Gene** | **Pearson R** | **P-Value** |
| --- | --- | --- |
| MMP7 | 0.2838 | < 0.0001 |
| NFATC1 | 0.2335 | < 0.0001 |
| PLCB3 | 0.2634 | < 0.0001 |
| RAC2 | 0.2119 | 0.0001 |
| RAC1 | 0.2183 | 0.0001 |
| CTBP2 | 0.1981 | 0.0003 |
| RHOA | 0.1945 | 0.0003 |
| PORCN | 0.1684 | 0.002 |
| FZD8 | 0.1631 | 0.0027 |
| FBXW11 | 0.1427 | 0.0088 |
| CHD8 | 0.1391 | 0.0107 |
| FOSL1 | 0.1228 | 0.0244 |
| SFRP4 | 0.1153 | 0.0347 |
| TBL1XR1 | 0.1125 | 0.0394 |
| CACYBP | 0.1102 | 0.0435 |

**Supplementary Methods**

**HCC cell lines and cell culture**

The human HCC cell lines, HepG2, Hep3B, and Sk-Hep1 were acquired from the American Type Culture Collection (Manassas, VA, USA). Huh7 cells were acquired from the Riken Cell Bank (Ibaraki, Japan). Bel-7402 cells were acquired from the Committee of Type Culture Collection of the Chinese Academy of Sciences (Shanghai, China). All cells were used within 15 passages and were cultured in RPMI 1640 supplemented with 10% fetal bovine serum in 5% CO2 at 37°C.

**Lentivirus vector construction and cell infection**

All the recombined lentivirus vector and the control vector were constructed by GenePharma (Shanghai, China). Lentiviral infection was performed by adding virus solution to Huh7 and Hep3B cells in the presence of 5μg/ml polybrene (Sigma-Aldrich, St. Louis, MO, USA). After infection for 72h, the cells were selected in the presence of 2μg/ml puromycin or 5μg/ml neomycin, and puromycin- or neomycin-resistant cells were collected and cultured. Construction of the stable cell lines was finished.

**Total RNA extraction and qPCR**

Total RNA was obtained from tissues or cells by using the TRIzol reagent (Invitrogen Corporation, Waltham, MA, USA) according to the manufacturer’s instructions. The concentration and purity of the RNA were assessed by using a NanoDrop 2000 instrument (Thermo Scientific, Waltham, MA, USA). First-strand cDNA was synthesized from total RNA by using a GoScript Reverse Transcription System (Promega, Madison, WI, USA). qPCR was performed using GoTaq qPCR Master Mix (Promega, Madison, WI, USA). *GAPDH* was used as an endogenous control for normalization.

**Tube formation assay**

Matrigel (BD, Franklin lake, New Jersey, USA) and serum-free RPMI-1640 were mixed in a ratio of 1:1 and then were added into a 96-well plate with 60μL mix per well. Centrifuge at 4°C for 5 min using 1800 rpm and then place the 96-well plate in 37°C for 30 min to solidify. 3×10^4^ HUVEC cells per well in 100ul supernatant collected from cells treated with different conditions for 72h were added to the 96-well plate. Culture for 3-6h at 37 °C and count the number of branches per well using a light microscope.

**Transwell migration assay**

Cells were harvested and seeded into the upper chambers (Corning, USA, 24-well plate, 8µm pores) at 5×10^4^ cells/well and serum free medium was added into the lower chambers. After 20h of incubation, cells on the upper inserts were wiped off carefully using cotton swabs. Cells that migrated through the filters were stained with 1% crystal violet for 30 min at room temperature and washed with PBS. The number of migrated cells was evaluated by counting all cells attached to the bottom of the inserts under a phase contrast microscope and quantified using the NIH Image-J software. The experiments were done in triplicate.

**Colony formation assay**

1×10^4^ HCC cells per well were added to the 6-well plate. Culture at 37°C for 14 days and fix with 4% polyformaldehyde. Stain with purple crystal and count the colony number using a light microscope.

**CCK8 assay**

1×10^3^ HCC cells per well in 100ul were added to the 96-well plate and cultured at 37°C. After 4 days’ culture, cell viability was assessed by CCK8 kit (Dojindu, Tokyo). A batch of cells was stained with 10mLof CCK8 regent at 37 for 1h and the absorbance at 450nm wavelength was measured to calculate the number of viable cells.

**Cell adhesion assay**

Fibronectin (10μg/mL, Sigma-Aldrich) was add to 96-well plate (60μl/well). Place the plate at 4 °C overnight for balance. Hydrate the plate using PBS and then block the plate using 2%BSA in PBS at 37°C for 30min. 2.5×10^4^ HCC cells per well in 100ul were added to the 96-well plate. Culture for 30min at 37°C.Then, count the number of adhesive cells by CCK8.

**Apoptosis assay**

After cell adherence, DMSO or sorafenib (20μg/mL, Sigma-Aldrich) was added the medium. After 48h culture at 37°C, cells were harvested and stained with ant-human Annexin V antibody (BD) and propidium iodide (BD) at 4°C. Analyze the apoptosis rate by flow cytometry in 15min.
